# New candidate genes for the fine regulation of the colour of grapes

**DOI:** 10.1093/jxb/erv159

**Published:** 2015-06-12

**Authors:** Laura Costantini, Giulia Malacarne, Silvia Lorenzi, Michela Troggio, Fulvio Mattivi, Claudio Moser, Maria Stella Grando

**Affiliations:** ^1^Department of Genomics and Biology of Fruit Crops, Research and Innovation Centre, Fondazione Edmund Mach, Via E. Mach 1, 38010 S. Michele all’Adige, Trento, Italy; ^2^Department of Food Quality and Nutrition, Research and Innovation Centre, Fondazione Edmund Mach, Via E. Mach 1, 38010 S. Michele all’Adige, Trento, Italy

**Keywords:** Anthocyanin, berry, candidate gene, metabolic profiling, microarray, quantitative trait locus, *Vitis vinifera.*

## Abstract

In the last decade, great progress has been made in clarifying the main determinants of anthocyanin accumulation in grape berry skin. However, the molecular details of the fine variation among cultivars, which ultimately contributes to wine typicity, are still not completely understood. To shed light on this issue, the grapes of 170 F_1_ progeny from the cross ‘Syrah’×’Pinot Noir’ were characterized at the mature stage for the content of 15 anthocyanins during four growing seasons. This huge data set was used in combination with a dense genetic map to detect genomic regions controlling the anthocyanin pathway both at key enzymatic points and at particular branches. Genes putatively involved in fine tuning the global regulation of anthocyanin biosynthesis were identified by exploring the gene predictions in the QTL (quantitative trait locus) confidence intervals and their expression profile during berry development in offspring with contrasting anthocyanin accumulation. New information on some aspects which had scarcely been investigated so far, such as anthocyanin transport into the vacuole, or completely neglected, such as acylation, is provided. These genes represent a valuable resource in grapevine molecular-based breeding programmes to improve both fruit and wine quality and to tailor wine sensory properties according to consumer demand.

## Highlight

The integration of metabolic, molecular marker, and transcriptomic data from a segregating grapevine progeny provides novel insights into the genetic control of anthocyanin content and composition in ripe berries.

## Introduction

Besides their direct role in the colour of grapes and wine, anthocyanins and their derived compounds also contribute to the sensory attributes of wine by interacting with molecules such as phenolics, polysaccharides, proteins, metals, and aroma substances (He *et al.*, 2012). Moreover, in recent years they have been reported to provide numerous beneficial effects for human health (Pojer *et al.*, 2013).

Anthocyanins are present in the skins of coloured cultivars as 3-monoglucosides of five anthocyanidins that are differently hydroxylated and *O*-methylated on the B ring. This chemical diversity may be further increased by acylation (aliphatic or aromatic) in the C6-position of the glucose moiety. These secondary modifications determine the chromatic properties of the pigments (Jackson, 2008). In particular, the hue is affected by the pattern of hydroxylation and methylation (a larger number of either free or methylated hydroxyl groups causes a bathochromic shift in the visible spectrum of anthocyanins) and by aromatic acylation (stacking of aromatic moieties of acylated anthocyanins plays a role in the blue colour shift). Colour intensity is promoted by acylation: as a general rule, acylated anthocyanins are preferentially trapped in anthocyanic vacuolar inclusions (AVIs), which has been proposed to result in further formation of AVIs and in dark coloration (Mizuno *et al.*, 2006).

The proportion of the different types of anthocyanins also has a large effect on pigment extractability and colour stability in winemaking, which in turn influence the wine profile. In particular, the non-acylated glucosides and acetyl-glucosides are more readily extracted from the fruit than the *p*-coumaroyl-glucosides, whose extraction rate in a wine-like solution is very low (Fournand *et al.*, 2006). With respect to pigment stability in grapes, the adjacent hydroxyl groups of *o*-diphenols in cyanidin, delphinidin, and petunidin enhance their susceptibility to oxidation. In contrast, due to methylation, neither malvidin nor peonidin possesses the ortho-positioned hydroxyl groups, which results in their comparatively higher resistance to oxidation (Jackson, 2008), a very desirable feature in winemaking. The acylation of the sugar can also promote anthocyanin chemical stability (Mazza *et al.*, 1995), as demonstrated in other plant species (Yonekura-Sakakibara *et al.*, 2009).

Finally, the different chemical structures of anthocyanins influence their bioavailability and bioactivity, with potential consequences on their health-promoting effects (Passamonti *et al.*, 2002; Rossetto *et al.*, 2002).

In the last decade, great progress has been made in clarifying the regulation of anthocyanin biosynthesis in grapevine (He *et al.*, 2010; Hichri *et al.*, 2011). The anthocyanin content in berry skin is mainly controlled by a cluster of *MYB*-type transcription factor genes on chromosome 2, with effects on both the qualitative distinction between coloured and white cultivars and the quantitative variation in anthocyanin level within the coloured varieties (Fournier-Level *et al.*, 2009; Huang *et al.*, 2013). However, grape accessions, even those displaying the same haplotype at the *MYBA* locus, show a continuous variation in anthocyanin content, which suggests the involvement of additional genes. For example, the products of *VvMYB5a*, *VvMYB5b*, *VvMYBPA1*, and *VvMYBPA2* have been proposed to modulate several genes in the common steps of the flavonoid pathway (Bogs *et al.*, 2007; Terrier *et al.*, 2009; Cavallini *et al.*, 2014). Moreover, in all model systems, the regulation of anthocyanin accumulation, both spatially and developmentally, results from a co-operative interaction between WD-repeat proteins, basic helix–loop–helix (bHLH) transcription factors, and MYB transcription factors, which form the MBW regulation complex. Recently, two bHLH and two WDR proteins were described in grapevine and, for one of them, VvMYC1, an involvement in the regulation of the flavonoid biosynthetic pathway was demonstrated (Hichri *et al.*, 2010; Matus *et al.*, 2010).

A few studies have looked into the variation in the relative abundance of specific anthocyanins, which is presumed to be finely tuned through tailoring reactions catalysed by flavonoid hydroxylases, glycosyltransferases, *O*-methyltransferases, and acyltransferases. In these studies, generally only one phenotype at a time was analysed (Jeong *et al.*, 2006; Falginella *et al.*, 2010; Fournier-Level *et al.*, 2011) or, when several traits were considered, a limited number of genes were investigated (Cardoso *et al.*, 2012). Acylation of anthocyanins has never been examined, although in some important cultivars the acylated anthocyanins can account for >60% of the total anthocyanins.

Finally, the complexity of skin colour might be regulated at the level of anthocyanin vacuolar sequestration. Transport into the vacuole is a prerequisite for anthocyanin biosynthesis and as such it fulfils a regulatory function (Marinova *et al.*, 2007), and it seems to be substrate specific (Mizuno *et al.*, 2006; Gomez *et al.*, 2009; Francisco *et al.*, 2013). Little is known about the exact mechanism of anthocyanin transportation in grapes. In addition to vesicle-mediated transport (Gomez *et al.*, 2011), several types of transporters have been proposed, most belonging to multigenic families which are little or not characterized in grapevine (for a review, see He *et al.*, 2010). Up to now, a few proteins were demonstrated to be involved in anthocyanin vacuolar sequestration in *Vitis vinifera*: a bilitranslocase homologue (Braidot *et al.*, 2008), a glutathione *S*-transferase (Conn *et al.*, 2008), two MATE-type (multidrug and toxic compound extrusion) proteins (Gomez *et al.*, 2009), and an ABC (ATP-binding cassette) transporter (Francisco *et al.*, 2013).

Very little is also known about anthocyanin degradation.

Here, the identification of new candidates for the regulation of anthocyanin accumulation in grape skin at technological maturity is described. To this end, a segregating progeny derived from the cross of two divergent varieties was analysed during four growing seasons for their anthocyanic profile, and an integrative approach combining metabolic, genetic, and transcriptional sources of information was adopted.

## Materials and methods

### Plant material

A population derived from ‘Syrah’×’Pinot Noir’ (clone ENTAV 115) consisting of 170 F_1_ individuals plus the parental lines was characterized at the biochemical, genetic, and transcriptomic level. All plants were grown at the experimental field Giaroni of FEM (Edmund Mach Foundation, San Michele all’Adige, Italy).

### Sampling

For anthocyanin analysis, two grape clusters were harvested from each plant at technological maturity (18 °Brix) in four different vintages (2007, 2008, 2009, and 2011) and stored at –80 °C till use. These samples were also analysed for flavonol content in a parallel experiment (Malacarne *et al.*, 2015). The number of phenotyped F_1_ individuals varied from 83 (in 2011) to 158 (in 2008). For microarray analysis, three individuals having very low anthocyanin content (LAPs: codes 256, 260, and PN) and three individuals having very high anthocyanin content (HAPs: codes 16, 56, and 63) were selected from the progeny. These six individuals number among eight divergent individuals for flavonol content (Malacarne *et al.*, 2015). The selection was statistically supported by analysis of variance (ANOVA) followed by Tukey HSD test (*P*-value <0.05) using Statistica 9 software (StatSoft, Tulsa, OK, USA). A representative sample of 50 berries harvested from both sides of the canopy was collected for each individual during the 2007 season at three berry developmental stages: pre-véraison (33E-L, hard green berry, PV), véraison (35E-L, 50% coloured berries, VER), and maturity (38E-L, 18 °Brix, MAT). From stage 35E-L, the collected berries were dissected into skin, flesh, and seeds, and then the skins were stored at –80 °C till transcriptomic analysis. These stages were chosen as véraison represents the starting point for anthocyanin accumulation in berry skin and maturity the stage at which anthocyanins were quantified in this work.

### Anthocyanin analysis

Anthocyanin extraction and HPLC-DAD (high-performance liquid chromatography-diode array detection) analysis were performed as described in Mattivi *et al.* (2006). The choice of methanol has a double justification: as a strong solvent it leads to the total extraction of the anthocyanins from the skins of grapes, which reduces the risk of differential extraction due to matrix effects; moreover, it avoids the partial hydrolysis of anthocyanin acetates that is observed with the widely used acidic solvents (Castia *et al.*, 1992). Fifteen anthocyanins were quantified by using a calibration curve with malvidin 3-glucoside chloride, and their content was expressed as the concentration (mg kg^–1^ of berries). Statistical analyses of biochemical data were done with the software SPSS 11.0 (IBM SPSS Statistics). The normality of trait distribution was checked by means of the Kolmogorov–Smirnov test. If necessary, data were log-transformed with a ln(1+x) function. Correlations between traits within years and between years within traits were determined using the non-parametric Spearman correlation coefficient.

### Expression analysis

RNA isolation, chip hybridization, and microarray data analysis were carried out as described at GEO (http://www.ncbi.nlm.nih.gov/geo/query/acc.cgi?acc=GSE42909) and in Malacarne *et al.* (2015), where the rationale of the approach is described in depth. Briefly, a total of 18 hybridizations (6 individuals×3 developmental stages) were performed on the custom Affymetrix GrapeGen GeneChip^®^ (Peng *et al.*, 2007). Probe sets with significant differential expression (DEPs) between LAPs and HAPs were selected in two steps under the assumption that the three individuals of each group can be treated as biological replicates (pseudoreplicates): (i) within each group (LAPs and HAPs) the data were analysed through pairwise comparisons of developmental stages (PV versus VER, PV versus MAT, and VER versus MAT). Differential expression analysis was performed with the Bayes *t*-statistics from LIMMA (Linear Models for Microarray Data), where *P*-values were corrected for multiple testing using the Benjamini–Hochberg method (Benjamini and Hochberg, 1995). Three lists of DEPs for each group were thus obtained [with a false discovery rate (FDR) <5% and a cut-off of 2-fold change (FC) between each pair of stages]; (ii) the lists of DEPs were then compared between LAPs and HAPs to provide a final list of probe sets either exclusively modulated in LAPs or in HAPs, or modulated in both groups but with low correlation (−0.5 > (logFC_LAPs_ – logFC_HAPs_)_pairwise comparison_ > 0.5).

Probe set annotation updated according to the 12Xv1 prediction (http://genomes.cribi.unipd.it/) and functional categorization were inferred from table S3 in Lijavetzky *et al.* (2012) and from the GrapeGen database (http://bioinfogp.cnb.csic.es/tools/GrapeGendb/).

Real-time reverse transcription–PCR (RT–PCR) analysis, carried out as described in Malacarne *et al.* (2015), was used (i) to validate microarray results technically; and (ii) to confirm the applicability of the pseudoreplicates approach by comparison with real biological replicates of each genotype.

### Map construction

Map construction was based on the segregation of 690 markers in 170 F_1_ individuals. Colour was mapped as a qualitative trait. SSR (simple sequence repeat) markers belonged to the series described in Supplementary Table S1 available at *JXB* online. SNP (single nucleotide polymorphism)-based markers were obtained from end sequences of BAC (bacterial artificial chromosome) clones used for Pinot Noir physical map construction (Troggio *et al.*, 2007) and from electronic SNPs identified after the completion of Pinot Noir genome sequencing (Pindo *et al.*, 2008). All the SSR and SNP markers were anchored to contigs included in the Pinot Noir physical map (Troggio *et al.*, 2007).

Markers were tested against the expected segregation ratio using a χ^2^ goodness-of-fit implemented in JoinMap 4.0 (Van Ooijen, 2006). Distorted markers were used for linkage analysis unless they affected the order of neighbouring loci. A consensus linkage map was generated by selecting LOD (log of odds) independence as the parameter in the grouping test (with a LOD threshold of 7.0) and regression mapping as the mapping algorithm. The Kosambi mapping function (Kosambi, 1944) was used to convert the recombination frequencies into map distances (cM, centiMorgans).

Linkage groups (LGs) were graphically visualized with MapChart 2.2 (Voorrips, 2002).

The accuracy of marker order estimated by meiotic methods was verified using the physical distance information from the BAC contigs with three or more mapped markers. For the sake of simplicity, base pairs (bp) were converted into cM by dividing by 300 000, which is the approximative physical length corresponding to 1 cM in grapevine (Velasco *et al.*, 2007).

### QTL analysis

QTL mapping was performed on the consensus map each year separately by using the simple interval mapping and multiple QTL mapping functions with the mixture model algorithm in MapQTL 6.0 (Van Ooijen, 2009). QTLs were declared significant if the maximum LOD exceeded the LG-wide LOD threshold (calculated using 1000 permutations) and mean error rate was <0.05. QTLs were considered reliable when consistently detected in at least two years. A non-parametric Kruskal–Wallis test was carried out to provide support to marker–trait associations. Confidence intervals corresponded to a LOD score drop of one on either side of the likelihood peak. Suffixes (a and b) were adopted to indicate different regions on the same LG where QTLs were found for anthocyanin (this work) and flavonol (Malacarne *et al.*, 2015) content. The 12X reference genome (http://genomes.cribi.unipd.it/) was used to extract version 1 of the gene predictions underlying QTLs.

A Fisher’s exact test was applied to evaluate the over-representation of specific functional categories in QTL regions controlling a given trait. Reference to the analysis was the distribution in the same categories of 30 748 12Xv1 gene predictions assigned to chromosomes (V1+V1r-ChrUn) as derived from Additional file 1 by Grimplet *et al.* (2012). Gene predictions annotated at the third level, as suggested by Mateos *et al.* (2002), were considered, with the exception of genes annotated at the first and second level if not characterized at a deeper level and significantly represented in the genome. Adjusted *P*-values obtained applying the Benjamini and Hochberg method (1995) for multiple testing correction were considered significant when ≤0.05.

### Candidate gene selection

Candidate genes were selected among the 12Xv1 predictions included in the confidence interval of reliable QTLs according to at least one of the following pieces of functional evidence: (i) literature supporting the involvement in trait regulation; (ii) differential expression between LAPs and HAPs (data from this study); (iii) expression detected in berry skin, possibly higher than in berry flesh (data from Fasoli *et al.*, 2012; Lijavetzky *et al.*, 2012), and consistent with the accumulation of anthocyanins during berry development; (iv) co-expression with genes involved in the regulation of anthocyanin biosynthesis or in flavonoid metabolism (data from COLOMBOS and VTC databases: Wong *et al.*, 2013; Meysman *et al.*, 2014); or (v) assignment to functional categories significantly over-represented in QTL regions (data from this study).

## Results and discussion

### Variation of anthocyanin content and composition in Syrah, Pinot Noir, and their progeny

**Fig. 1.**
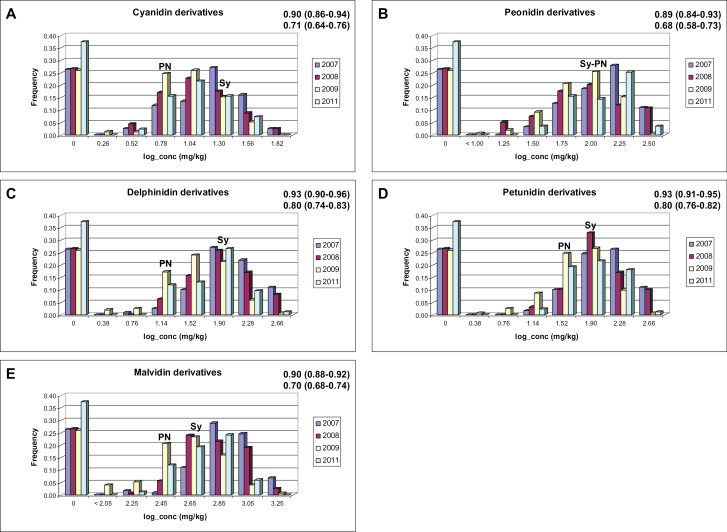
Variation of anthocyanin content in the Syrah×Pinot Noir progeny in four different vintages. For the two parents, averaged values across years are reported. In the right upper part of each plot, the Spearman rank-order correlation between years (mean value and range of variation in parentheses) is shown for the whole progeny (top) and the coloured progeny (bottom). Correlations are significant at the 0.01 level. Sy, Syrah; PN, Pinot Noir; conc, concentration. (This figure is available in colour at *JXB* online.)

A detailed description is presented in Fig. 1, and Supplementary Text S1, Tables S2–S4, and Fig. S1 at *JXB* online.

### Map construction

**Fig. 2.**
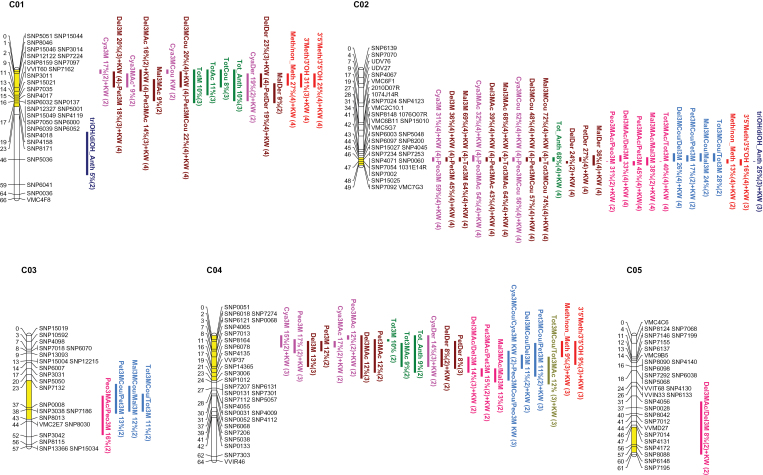

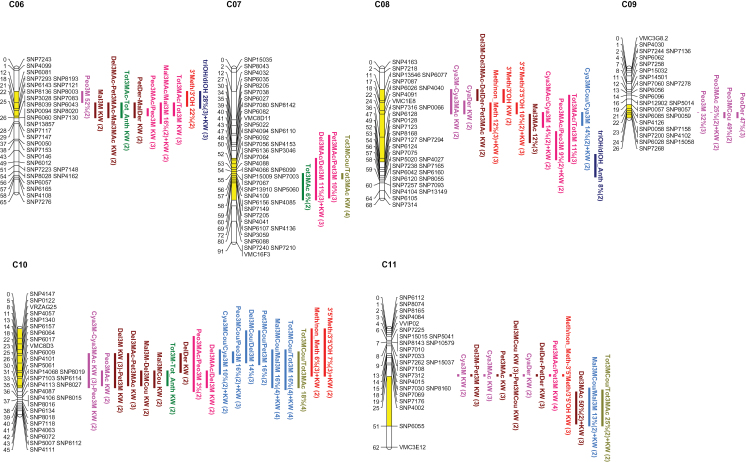

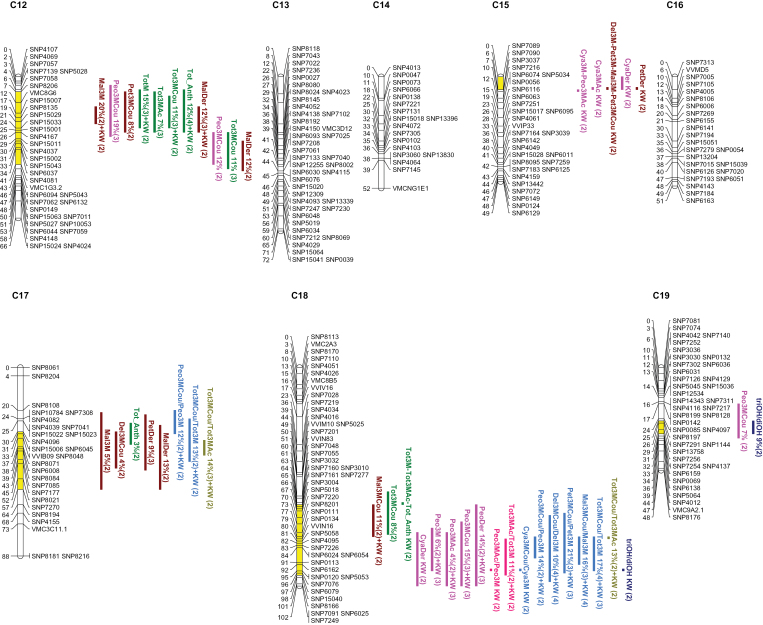
Linkage map of the Syrah×Pinot Noir cross. Distances of markers from the top (in cM) are indicated on the left side of linkage groups. Bars on the right correspond to 1–LOD confidence intervals of QTLs identified in at least two seasons (or to regions where significant markers were found with Kruskal–Wallis analysis). For each trait, the average phenotypic variance explained by the QTL and the number of years (in parentheses) in which significants QTLs (or markers) were detected are reported. The marked portion of the linkage group corresponds to the overall confidence interval used for gene prediction extraction. Cya, cyanidin; Peo, peonidin; Del, delphinidin; Pet, petunidin; Mal, malvidin; 3M, 3-monoglucoside; Ac, acetate; Cou, *p*-coumarate; Tot, total; Der, derivatives (3-monoglucoside+3-monoglucoside-acetate+3-monoglucoside-*p*-coumarate); 3ʹMeth/3ʹOH, peonidin 3-monoglucoside/cyanidin 3-monoglucoside; 3ʹ5ʹMeth/3ʹ5ʹOH, malvidin 3-monoglucoside/delphinidin 3-monoglucoside; diOH, di-hydroxylated (cyanidin+peonidin 3-monoglucoside); triOH, tri-hydroxylated (delphinidin+petunidin+malvidin 3-monoglucoside). (This figure is available in colour at *JXB* online.)

Out of the 690 scored markers, 36 were discarded as they had too much missing data, they were identical to other loci, or their genetic position was inconsistent with the physical position. Out of the 654 remaining markers, 593 could be finally placed onto 19 LGs covering 1184 cM with an average distance between markers of 2 cM (Fig. 2; Supplementary Table S1 at *JXB* online). QTL analysis is expected to benefit from it, as high-density maps were recently demonstrated to improve the precision of QTL localization and effect estimation, especially for minor QTLs, as well as the power to resolve closely linked QTLs (Stange *et al.*, 2013).

### QTL analysis

Thanks to the generation of an improved genetic map of Syrah×Pinot Noir and of chemical data collected over four growing seasons, several consistent QTLs for the content of each compound, for their sums, and for the ratios between specific classes of anthocyanins could be identified, with different effects and stability among years (Fig. 2; Supplementary Table S5 at *JXB* online).

By analysing all the progeny, a major QTL on LG 2 was detected for all the 15 anthocyanins and for their sums (‘totals’), which explained between 31% and 74% of phenotypic variance averaged over years. When considering only the coloured F_1_ individuals, the same QTL was found to regulate most of the above traits as well as their sums (‘derivatives’) and ratios with a percentage of explained variance ranging from 6% to 45%. As a general trend, in the coloured progeny, the effect of this QTL was lower or lacking for traits involving dihydroxylated anthocyanins. Candidate genes for this QTL were previously identified in a cluster of MYB-type transcription factors genes (Fournier-Level *et al.*, 2009).

Several smaller effect QTLs were detected on LGs 1, 3, 4, 5, 6, 7, 8, 9, 10, 11, 12, 17, 18, and 19, most of which were never reported before. Only a few regulated a specific trait (e.g. the peonidin level in the case of the QTL on LG 9), while many of them showed pleiotropic effects on several traits. However, in some cases, a primary effect could be argued, which then gives rise to a number of secondary effects. For example, it is likely that the QTL on LG 1a primarily regulates the methylation level of anthocyanins (25–31% of explained variance), in agreement with the findings of Fournier-Level *et al.* (2011), who identified the underlying molecular factors in an anthocyanin-*O*-methyltansferase gene cluster. Similarly, it can be proposed that the QTL on LG 6 controls the hydroxylation level of anthocyanins (28% of explained variance) and that the QTLs on LGs 3, 7b, 8b, 12b, and 18b contribute to the acylation level of anthocyanins (6–21% of explained variance). In contrast, the QTLs identified on LGs 4b, 5, 8a, 10, 11a, 12a, and 17 might be related to a more general activity since they seem to regulate flavonol content and composition as well (Malacarne *et al.*, 2015). Several QTLs (in particular those identified on LGs 2, 6, 8a, and 17) also co-localize with regions controlling proanthocyanidin content and composition (Huang *et al.*, 2012). Finally, the QTL identified on LG 8b coincides with a QTL explaining up to 32% of variance for total anthocyanin content in interspecific hybrid grapes (Ban *et al.*, 2014).

### Candidate gene identification

The number of genes within QTL confidence intervals varied from a minimum of 149 (LG 2) to a maximum of 861 (LG 8) (Table 1).

**Table 1.** Genes with a potential role in the regulation of anthocyanin content and composition No symbol is shown at the side of gene predictions that are not represented on the GrapeGen GeneChip, whereas * and # indicate the presence and absence of differential expression for gene predictions that are represented on the GrapeGen GeneChip, respectively. The column named ‘This study’ indicates whether a candidate gene is novel (i.e. no previous evidence was available).

**Candidate gene**	**Functional evidence**	**This study**
*LG 1a: 0.0–18.8 cM=10 284 104–23 021 414bp (636 gene predictions*)		
Caffeoyl-CoA *O*-methyltransferase: VIT_01s0010g03460, VIT_01s0010g03470 (*VvOMT3*), VIT_01s0010g03490 (*VvOMT2*), VIT_01s0010g03510* (*VvOMT1*)	Anthocyanin methylation affected by transcriptional regulation of *VvOMT1* and by structural variation of *VvOMT2* (Fournier-Level *et al.*, 2011) *VvOMT1* highly induced at véraison in both HAPs and LAPs, with a slightly higher fold change in HAPs; more expressed in the skin than in the flesh; co-expressed with *VvMYBA1*	
Glutathione *S*-transferase: VIT_01s0026g01340#, VIT_01s0026g01370, VIT_01s0026g02400*	*AtGSTU17* and *ZmBz2* co-expressed with the flavonoid biosynthetic genes and responsible for the transport of anthocyanins into the vacuole (Marrs *et al.*, 1995; Yonekura-Sakakibara *et al.*, 2008); VIT_01s0026g01340 more expressed in the skin than in the flesh VIT_01s0026g01370 induced by cluster thinning, in parallel with an increase in anthocyanins (Pastore *et al.*, 2011) VIT_01s0026g02400 induced at maturity in both HAPs and LAPs; more expressed in the skin than in the flesh	X
Calcium-binding protein CML: VIT_01s0026g02590*	Induced at maturity in both HAPs and LAPs, with a higher fold change in HAPs	X
*LG 1b: 22.8–59.0 cM (not analysed*)		
*LG 2: 45.3–49.5 cM=14 047 004–18 251 419bp (149 gene predictions*)		
MYB domain protein: VIT_02s0033g00370 (*VvMYBA4*), VIT_02s0033g00380, VIT_02s0033g00390* (*VvMYBA2*), VIT_02s0033g00410* (*VvMYBA1*), VIT_02s0033g00430, VIT_02s0033g00440, VIT_02s0033g00450* (*VvMYBA3*)	Polymorphisms in *VvMYBA1*, *VvMYBA2*, and *VvMYBA3* associated with the quantitative variation in anthocyanin content (Fournier-Level *et al.*, 2009) *VvMYBA1*, *VvMYBA2*, and *VvMYBA3* induced at véraison in both HAPs and LAPs, with a higher fold change in HAPs *VvMYBA1* more expressed in the skin than in the flesh Co-expressed	
Alternative oxidase 1D: VIT_02s0033g01380*	Involved in ABA signalling, induced at maturity in both HAPs and LAPs, with a higher fold change in HAPs; more expressed in the skin than in the flesh	X
Ankyrin repeat family: VIT_02s0087g00100#	AtPIA2 (PHYTOCHROME INTERACTING ANKYRIN-REPEAT PROTEIN 2) mediating the interaction between phytochrome A and partner proteins to regulate the expression of anthocyanin biosynthetic genes (Yoo *et al.*, 2011)	X
*LG 3: 22.7–43.1 cM=3 433 730–6 688 642bp (329 gene predictions*)		
Serine carboxypeptidase: VIT_03s0063g00860#	Co-expressed with *VvMYBA1*	X
Glucosyltransferase: VIT_03s0091g00040# (*VvGT1*), VIT_03s0180g00200# (*VvGT2*), VIT_03s0180g00320# (*VvGT3*)	At4g15480 belonging to an anthocyanin regulon (Vanderauwera *et al.*, 2005) Recombinant VvGT1–VvGT3 enzymes catalysing the formation of acyl-glucose esters of phenolic acids, which could act as acyl donors in esterification reactions by SCPL proteins (Khater *et al.*, 2012); more expressed in the skin than in the flesh; *VvGT1* induced by cluster thinning, in parallel with an increase in anthocyanins (Pastore *et al.*, 2011)	
Cinnamyl alcohol dehydrogenase: VIT_03s0180g00260*	Induced at maturity only in HAPs	
*LG 4b: 0.0–23.0 cM=16 020 906–23 862 496bp (577 gene predictions*)		
Calmodulin: VIT_04s0023g01100*	Induced at maturity only in HAPs	X
Flavonone-3-hydroxylase: VIT_04s0023g03370*	More expressed in the skin than in the flesh; up-regulated in *VlMybA1-2*-transformed hairy roots (Cutanda-Perez *et al.*, 2009) and by low nitrogen supply, in parallel with an increase in anthocyanins (Soubeyrand *et al.*, 2014); co-expressed with genes significantly enriched in the functional category ‘Phenylpropanoid biosynthetic process’	
SEN1 (dark inducible 1): VIT_04s0023g03600*	Involved in jasmonate signalling, induced at véraison only in HAPs	X
*LG 5: 47.4–60.1 cM=745 680–4 176 378bp (373 gene predictions*)		
Receptor-like protein kinase: VIT_05s0020g00730*	Reduced at maturity only in LAPs; more expressed in the skin than in the flesh	X
BZIP protein HY5-HOMOLOG (HYH): VIT_05s0020g01090#	AtHY5/HYH involved in light signalling and regulating low temperature-induced anthocyanin accumulation (Zhang *et al.*, 2011) VIT_05s0020g01090 more expressed in the skin than in the flesh; up-regulated by UV radiation in berry skin, in parallel with an increase in some acylated anthocyanins (Carbonell-Bejerano *et al.*, 2014*a* ); co-expressed with genes significantly enriched in the functional category ‘Anthocyanidin 3-*O*-glucosyltransferase activity’	X
*LG 6: 12.2–25.6 cM=12 767 560–17 912 115bp (265 gene predictions*)		
Calmodulin-related protein: VIT_06s0009g01920*	Reduced during development only in LAPs; potentially involved in the calmodulin- dependent signal transduction pathways that mediate sugar induction of anthocyanin synthesis (Vitrac *et al.*, 2000); up-regulated by higher sugar, in parallel with an increase in total anthocyanins and in the delphinidin/cyanidin derivative ratio (Dai *et al.*, 2014)	X
Flavonoid 3ʹ,5ʹ-hydroxylase: VIT_06s0009g02830*, VIT_06s0009g02840, VIT_06s0009g02910, VIT_06s0009g03010*	Proportion of delphinidin- and cyanidin-based anthocyanins correlating with the ratio of *F3ʹ5ʹH* to *F3ʹH* expression (Jeong *et al.*, 2006; Falginella *et al.*, 2010); VIT_06s0009g02830 and VIT_06s0009g03010 induced at véraison in both HAPs and LAPs, with a slightly higher fold change in HAPs; VIT_06s0009g03010 more expressed in the skin than in the flesh; VIT_06s0009g02910 and VIT_06s0009g03010 induced by cluster thinning, in parallel with an increase in anthocyanins (Pastore *et al.*, 2011); VIT_06s0009g02840 induced by low nitrogen supply, in parallel with an increase in anthocyanins (Soubeyrand *et al.*, 2014); significant enrichment of the functional category ‘Metabolism.Secondary metabolism.Phenylpropanoid metabolism’	
Hexokinase 6: VIT_06s0061g00040	Potentially involved in sugar signalling transduction leading to anthocyanin accumulation (Vitrac *et al.*, 2000); up-regulated by higher sugar, in parallel with an increase in total anthocyanins and in the delphinidin/cyanidin derivative ratio (Dai *et al.* 2014)	X
*LG 7b: 46.6–69.3 cM=4 252 958–14 369 584 bp (500 gene predictions*)		
ABC transporter C member 9: VIT_07s0005g04680	Induced by cluster thinning, in parallel with an increase in anthocyanins (Pastore *et al.*, 2011); significant enrichment of the functional category ‘Transport overview. Macromolecule transport.Multidrug transport’	X
Glutathione *S*-transferase 25 GSTU7: VIT_07s0005g04890*	Induced at maturity in both HAPs and LAPs; co-expressed with the *VvMYBA* genes on chromosome 2; significant enrichment of the functional category ‘Transport overview. Macromolecule transport.Multidrug transport’	X
LG 8a: 11.5–24.1 cM=17 261 077–20 013 908 bp (283 gene predictions)		
Anthocyanin membrane protein 1 (Anm1): VIT_08s0007g03560, VIT_08s0007g03570*	VIT_08s0007g03560 co-expressed with *VvMYBA1* VIT_08s0007g03570 induced at véraison in both HAPs and LAPs, with a higher fold change in HAPs; more expressed in the skin than in the flesh	X
4-Coumarate-CoA ligase: VIT_08s0007g05050	Induced by cluster thinning, in parallel with an increase in anthocyanins (Pastore *et al.*, 2011)	
*LG 8b: 24.1–48.5 cM=11 523 362–17 250 890bp (578 gene predictions*)		
AUX1 protein: VIT_08s0007g02030*	AtAUX1 involved in anthocyanin accumulation in response to UV light VIT_08s0007g02030 reduced during development only in LAPs; more expressed in the skin than in the flesh	X
ATAN11 (ANTHOCYANIN11): VIT_08s0007g02920#	PhAN11 involved in the post-translational regulation of the MYB-domain transcriptional activator *PhAN2* (de Vetten *et al.*, 1997) VIT_08s0007g02920 more expressed in the skin than in the flesh	X
P-coumaroyl shikimate 3ʹ-hydroxylase isoform 1: VIT_08s0040g00780*	Induced at maturity only in HAPs; more expressed in the skin than in the flesh; co-expressed with genes significantly enriched in the functional category ‘Phenylpropanoid biosynthetic process’	
Serine carboxypeptidase: VIT_08s0040g01040, VIT_08s0040g01060#, VIT_08s0040g01110	Specifically up-regulated around véraison (Sweetman *et al.*, 2012)	X
Phenylalanine ammonia-lyase: VIT_08s0040g01710*	Induced at maturity in both HAPs and LAPs, with a slightly higher fold change in HAPs; more expressed in the skin than in the flesh	
Calcium-transporting ATPase 1, endoplasmic reticulum-type ECA1: VIT_08s0040g02830*	Induced at maturity only in HAPs; Ca^2+^ ATPases involved in anthocyanin accumulation in carrot callus cultures (Sudha and Ravishankar, 2003)	X
Glutathione *S*-transferase GSTO1: VIT_08s0040g03040*	Induced at maturity (one probe exclusively in HAPs); induced by cluster thinning, in parallel with an increase in anthocyanins (Pastore *et al.*, 2011)	X
TTG2 (TRANSPARENT TESTA GLABRA 2): VIT_08s0040g03070# (*VvWRKY19*)	AtTTG2 regulating proanthocyanidin and possibly anthocyanin production (Ishida *et al.*, 2007) VvWRKY19 proposed as an intermediate regulator of flavonoid biosynthetic pathway acting downstream of *VvMYB5a* and *VvMYB5b* and in concert with the regulatory complex MBW, with an effect on anthocyanin accumulation among others (Cavallini, 2012); more expressed in the skin than in the flesh	
*LG 8c: 44.9–57.0 cM (not analysed*)		
*LG 9: 19.0–26.1 cM=13 924 117–19 956 861bp (202 gene predictions*)		
WRKY DNA-binding protein 40: VIT_09s0018g00240*	Induced at maturity in both HAPs and LAPs, with a slightly higher fold change in HAPs; more expressed in the skin than in the flesh	X
*LG 10: 0.0–30.7 cM=17 991–9 015 334bp (605 gene predictions*)		
Squamosa promoter-binding protein 3 (SPL3): VIT_10s0003g00050*	*LeSPL-CNR* residing at at the tomato Colorless non-ripening (Cnr) locus and showing reduced expression in the colourless mutant (Manning *et al.*, 2006) VIT_10s0003g00050 reduced at maturity only in LAPs; more expressed in the skin than in the flesh	X
Protein kinase: VIT_10s0003g01420*, VIT_10s0003g03330	VIT_10s0003g01420 induced at maturity only in HAPs; VIT_10s0003g03330 co-expressed with genes significantly enriched in the functional category ‘Flavonoid metabolic process’; significant enrichment of the functional category ‘Signaling. Signaling pathway.Protein kinase’	X
Heat shock transcription factor A4A: VIT_10s0003g01770#	Associated with berry colour (Ageorges *et al.*, 2006)	
Multidrug resistance-associated protein 5: VIT_10s0003g03470	Induced by cluster thinning, in parallel with an increase in anthocyanins (Pastore *et al.*, 2011)	X
Jasmonate ZIM domain-containing protein 8: VIT_10s0003g03790*	Induced during development only in LAPs, with a high fold change	X
Early-responsive to dehydration stress protein (ERD4): VIT_10s0003g04180*	Induced at véraison only in HAPs	X
WRKY DNA-binding protein 6: VIT_10s0116g01200*	Induced at maturity in both HAPs and LAPs, with a slightly higher fold change in HAPs; more expressed in the skin than in the flesh	X
Auxin-responsive protein: VIT_10s0405g00040*	Induced at véraison only in HAPs, with a high fold change; more expressed in the skin than in the flesh	X
*LG 11a: 25.1–51.0 cM=12 199–5 477 250bp (558 gene predictions*)		
MYB domain protein: VIT_11s0016g01300 (*VvMYBPAR*), VIT_11s0016g01320# (*VvMYBPA2*)	Regulating proanthocyanidin (PA) biosynthesis through activation of general flavonoid pathway genes and of PA-specific genes (Terrier *et al.*, 2009; Koyama *et al.*, 2014); *VvMYBPA2* more expressed in the skin than in the flesh	
Clavata1 receptor kinase (CLV1): VIT_11s0016g03080*	Induced during development in both HAPs and LAPs, with a higher fold change in HAPs; more expressed in the skin than in the flesh	X
*LG 12a: 7.3–26.2 cM=1 682 201–5 318 476bp (317 gene predictions*)		
Ethylene-responsive transcription factor 9: VIT_12s0028g03270*	Induced during development only in HAPs; more expressed in the skin than in the flesh; co-expressed with *VvMYBA1*	X
*LG 12b: 26.2–40.5 cM=5 320 497–9 268 609bp (358 gene predictions*)		
Auxin-responsive protein AIR12: VIT_12s0057g00420*	AtAIR12 involved in positive regulation of the flavonoid biosynthetic process VIT_12s0057g00420 induced at maturity in both HAPs and LAPs, with a higher fold change in HAPs; more expressed in the skin than in the flesh; co-expressed with genes significantly enriched in the functional category ‘Ammonia-lyase activity’	X
Protein kinase: VIT_12s0057g00520, VIT_12s0134g00450*	VIT_12s0057g00520 co-expressed with genes significantly enriched in the functional category ‘Regulation of anthocyanin metabolic process’, including the *VvMYBAs* on chromosome 2 VIT_12s0134g00450 induced at véraison only in HAPs; more expressed in the skin than in the flesh; co-expressed with genes significantly enriched in the functional category ‘Flavonoid metabolic process’ Significant enrichment of the functional category ‘Signaling.Signaling pathway.Protein kinase’	X
Prephenate dehydratase: VIT_12s0059g00750#	More expressed in the skin than in the flesh; co-expressed with *VvMYBA1*	
Acyltransferase: VIT_12s0134g00580 (*VvAT1*), VIT_12s0134g00590 (*VvAT2*), VIT_12s0134g00600 (*VvAT3*), VIT_12s0134g00620 (*VvAT4*), VIT_12s0134g00630 (*VvAT5*), VIT_12s0134g00640 (*VvAT6*), VIT_12s0134g00650# (*VvAT7*), VIT_12s0134g00660 (*VvAT8*),VIT_12s0134g00670# (*VvAT9*)	VIT_12s0134g00590 up-regulated specifically in the berry skin during the daytime near maturity (Carbonell-Bejerano *et al.*, 2014*b* ) VIT_12s0134g00620 induced by cluster thinning, in parallel with an increase in anthocyanins (Pastore *et al.*, 2011) VIT_12s0134g00670 more expressed in the skin than in the flesh Significant enrichment of the functional category ‘Metabolism.Secondary metabolism. Phenylpropanoid metabolism’	X
*LG 17: 30.1–57.5 cM=4 031 362–9 700 367bp (430 gene predictions*)		
COBRA-like protein 4: VIT_17s0000g05050*	AtCOB proteins playing a role in anthocyanin synthesis (Ko *et al.*, 2006) VIT_17s0000g05050 reduced at véraison only in LAPs; proposed as a regulator of proanthocyanidin total quantity (Carrier *et al.*, 2013)	
Myb domain protein 94: VIT_17s0000g06190*	*ZmMYB31* overexpression leading to a large increase in the anthocyanin level through the up-regulation of anthocyanin biosynthetic genes (Fornalé *et al.*, 2010) VIT_17s0000g06190 induced at véraison only in HAPs; more espressed in the skin than in the flesh; paralleling the kinetics of anthocyanin accumulation during ripening (Ziliotto *et al.*, 2012)	X
Flavonoid 3ʹ-hydroxylase: VIT_17s0000g07210#	More expressed in the skin than in the flesh; induced by cluster thinning, in parallel with higher relative levels of dihydroxylated anthocyanins (Pastore *et al.*, 2011)	
EDS1 (Enhanced disease susceptibility 1): VIT_17s0000g07560*	Involved in jasmonate signalling, induced at maturity only in HAPs; more expressed in the skin than in the flesh	X
*LG 18b: 64.0–97.4 cM=16 285 312–28 018 133bp (566 gene predictions*)		
UDP-glucose:anthocyanidin 5,3-*O*-glucosyltransferase: VIT_18s0041g00710#, VIT_18s0041g00740*, VIT_18s0041g00840, VIT_18s0041g00950	VIT_18s0041g00710 more expressed in the skin than in the flesh; VIT_18s0041g00740 induced at maturity in both HAPs and LAPs; VIT_18s0041g00840 and VIT_18s0041g00950 induced by cluster thinning, in parallel with an increase in anthocyanins (Pastore *et al.*, 2011); significant enrichment of the functional category ‘Metabolism.Secondary metabolism.Phenylpropanoid metabolism’	X
Peroxidase 12: VIT_18s0072g00160*	Induced at maturity in both HAPs and LAPs, with a higher fold change in HAPs; more expressed in the skin than in the flesh	
Leucine-rich repeat protein kinase: VIT_18s0072g00990*	Induced at maturity only in HAPs; more expressed in the skin than in the flesh	X
*LG 19: 25.4–30.8 cM=4 694 353–9 176 259bp (397 gene predictions*)		
Avr9/Cf-9 rapidly elicited protein 20: VIT_19s0014g04650*	At4g27280 up-regulated after *PAP1* overexpression and probably involved in the downstream regulation of anthocyanin biosynthesis by *PAP1* (Tohge *et al.* 2005) VIT_19s0014g04650 induced at maturity only in HAPs	X
ABC transporter C member 9: VIT_19s0015g00010 (*VvMRP2*), VIT_19s0015g00040 (*VvMRP4*), VIT_19s0015g00050# (*VvMRP5* in Cakir *et al.*, 2013)	VIT_19s0015g00010 and VIT_19s0015g00040 induced by cluster thinning, in parallel with an increase in anthocyanins (Pastore *et al.*, 2011); VIT_19s0015g00050 more expressed in the skin than in the flesh; significant enrichment of the functional category ‘Transport overview.Macromolecule transport.Multidrug transport’	X

Without claiming to be exhaustive, candidate genes for trait regulation were identified according to the criteria described in the Materials and methods (expression data and enrichment tests are reported in Supplementary Tables S6 and S7, respectively, at *JXB* online). Candidate functions based on previous knowledge were: regulation of transcription, enzymatic activity, transport, and signalling/response to stimulus. Among structural enzymes, those involved in phenylalanine, phenylpropanoid, flavonoid, and anthocyanin metabolism were evaluated. Candidate enzymes for anthocyanin degradation, such as peroxidases, laccases, polyphenol oxidases, and β-glucosidases (Oren-Shamir, 2009), were also considered. Among transport-related molecules, priority was given to GST (mainly of class phi; Dixon et al, 2010), ABC (ABCC subfamily; Cakir *et al.*, 2013), and MATE proteins, based on the crucial role they play in anthocyanin vacuolar sequestration in *Arabidopsis* and several crops, including grapevine (Zhao and Dixon, 2009). These proteins are abundant in plants, especially in grapevine, for which 87, 26, and 65 genes were predicted for the most important classes of GST, ABC, and MATE proteins, respectively (Gomez *et al.*, 2009; Zenoni *et al.*, 2010; Cakir *et al.*, 2013). Therefore, their co-localization with QTLs is expected to help in the selection of candidates for functional characterization. Indeed, sequence similarity has not always proved to be sufficient to predict functional similarity (Alfenito *et al.*, 1998). Similarly, a strict correlation between the expression of transport-related proteins and anthocyanin accumulation during berry ripening is not expected in view of gene redundancy in plant transporter families on the one hand and their multifunctionality and up-regulation effect by their substrates on the other hand. Finally, genes involved in response to stimuli which are known to affect anthocyanin content and composition were taken into account. In particular, anthocyanin accumulation is considered a common response to stress conditions (Teixeira *et al.*, 2013). Sugars also induce anthocyanin biosynthesis through the participation of hexokinases, Ca^2+^, calmodulin, protein kinases, and protein phosphatases (Vitrac *et al.*, 2000; Lecourieux *et al.*, 2014). As regards hormones, abscisic acid, auxin, cytokinins, ethylene, and salicylic acid are typically responsible for an increase in anthocyanins, whereas gibberellic acid has the opposite effect. Methyl jasmonate, which is a stress signalling molecule, has been shown to elicit a higher level of anthocyanins, whereas JASMONATE-ZIM domain (JAZ) proteins repress anthocyanin accumulation (He *et al.*, 2010; Qi *et al.*, 2011).

In-house expression data for progeny individuals with contrasting anthocyanin content (LAPs and HAPs) were used to support the choice of candidates genes. The reliability of these data was confirmed by real-time RT–PCR (Supplementary Fig. S2 at *JXB* online; Malacarne *et al.*, 2015). Comparative analysis of the transcriptome at three developmental stages for each triplet of extreme individuals revealed the differential expression of 3755 and 2933 probe sets in LAPs and HAPs, respectively. Of these, 1841 were exclusively modulated in LAPs and 1019 in HAPs, while 1914 were commonly modulated but with low correlation (Supplementary Table S6). This approach was preferred to the direct comparison between LAPs and HAPs at each developmental stage in order to minimize the possible differences due to non-perfect phase sampling.

The selected candidate genes are shown in Table 1. Some previous findings have been confirmed, with the addition of new information. For example, the co-localization of the QTLs for 3ʹ and 5ʹ methylation (Fig. 2; Supplementary Table S5 at *JXB* online) and the high correlation between the 3ʹMeth/3ʹOH and 3ʹ5ʹMeth/3ʹ5ʹOH ratios in the Syrah×Pinot Noir progeny (Suppelmentary Table S4A) and in the grapevine germplasm (Mattivi *et al.*, 2006) suggest that the candidate enzymes are able to perform both reactions, as biochemically demonstrated for the methyltransferase that best matches with VvAOMT1 (Hugueney *et al.*, 2009). The identification of a QTL regulating the hydroxylation level on LG 6 supports the involvement of the flavonoid 3ʹ,5ʹ-hydroxylases that have been previously suggested based on expression analysis (Jeong *et al.*, 2006; Falginella *et al.*, 2010), providing a possible genetic determination for trait variation (in contrast, gene expression might be the result of a cascade). Most of the candidate genes shown in Table 1 are reported for the first time and represent a valuable resource for functional characterization.

### Anthocyanin acylation

One of the objectives of this work was a better understanding of the acylation step, which is, at the moment, completely obscure in grapevine. Unfortunately, no QTL could be found for the presence/absence of acylated anthocyanins, as expected by lack of segregation for this Pinot Noir trait in the progeny. However, several minor QTLs, which might regulate the quantitative variation in the acylation level, were identified (Fig. 2; Supplementary Table S5 at *JXB* online). The strongest candidate genes code for enzymes responsible for anthocyanin decoration with acyl groups, which can be classified within the BAHD and serine carboxypeptidase-like (SCPL) families (St-Pierre *et al.*, 2000; Milkowski and Strack, 2004). For example, in the confidence interval of the QTL on LG 12b, which explains ~12% of the phenotypic variance for peonidin-3-monoglucoside-*p*-coumarate and malvidin derivatives, a cluster of nine BAHD acyltransferases was found. After screening for the presence of conserved motifs, two of them (*VvAT1* and *VvAT9*) were retained for phylogenetic analysis and were grouped in clade Ia with plant acyltransferases involved specifically in the modification of anthocyanins. In contrast, the remaining grapevine gene predictions considered for phylogenetic analysis were attributed to clades Ib–Vb, whose members are specific to different substrates (Supplementary Fig. S3). As for methylation and hydroxylation, a QTL controlling several acylation-related subtraits, which co-localizes with the cluster of *MYBA* transcription factors, was detected on LG 2 (Fig. 2; Supplementary Table S5). The *MYBA* locus might exert its pleiotropic effect on these traits indirectly (as a consequence of its effect on total anthocyanin accumulation); however it is tempting to speculate that grapevine anthocyanin acyltransferase genes are regulated by MYB factors, similar to what was reported in *Arabidopsis* and *Gerbera hybrida* (Tohge *et al.*, 2005; Laitinen *et al.*, 2008). In agreement with this hypothesis, a very high proportion of coumaroylated anthocyanins (70%) was found in Syrah roots overexpressing *VlMYBA1-2*, while ~30% of this kind of anthocyanins are usually found in mature red berries from the same cultivar (Cutanda-Perez *et al.*, 2009).

Alternative candidates for the QTL on LG 18b, which putatively explains from 10% to 21% of the phenotypic variance related to acylation (Fig. 2; Supplementary Table S5 at *JXB* online), are a cluster of UDP-glucose:anthocyanidin 5,3-*O*-glucosyltransferases, mostly expressed in *V. vinifera* berry skin (Fasoli *et al.*, 2012). It is not known whether these enzymes really perform two sequential glucosylations as suggested by their annotation, considering that 3-*O*-glucosylation is generally required and sufficient for anthocyanidin stabilization in *V. vinifera*. However, it can be speculated that they glucosylate anthocyanins, and glucosylated anthocyanins are the substrates for acyltransferases. The presence on LG 18b of a gene cluster comprising 29 predictions for laccase is also noteworthy. Most of them were reported to be expressed in berry skins, especially at post-harvest (Fasoli *et al.*, 2012), which is in agreement with the higher degradation rate observed at over-ripening for coumaroylglucoside derivatives than for non-acylated glucoside derivatives or acetylglucoside derivatives (Fournand *et al.*, 2006). Finally, a significant enrichment of QTL regions related to the content of *p*-coumaric derivatives was observed in gene predictions belonging to the functional subcategories ‘Response to stimulus.Stress response.Biotic stress response’ and ‘Diverse functions.Gene family with diverse functions.NBS-LRR superfamily’ (for the QTLs on LG 18b) and ‘Response to stimulus.Stress response.Abiotic stress response’ (for the QTL on LG 12b) (Supplementary Table S7). This observation is in agreement with the integrative analysis of Zamboni *et al.* (2010) showing a correlation of coumarated anthocyanins, that are accumulated mainly during withering, with stress- and pathogenesis-induced transcripts and proteins.

### Conclusions

In this work, a huge amount of data has been generated to characterize the genetic bases of anthocyanin content and composition in ripe grapevine berries. A dense map was obtained from the segregation of ~600 markers, and the amount of 15 distinct anthocyanins was measured during four growing seasons in the Syrah×Pinot Noir progeny. Several consistent QTLs were identified and special attention was paid to the minor ones, which might be responsible for the fine regulation of berry and wine colour. Based on expression data from this study and on functional evidence from the literature, reliable candidate genes are proposed, which represent a valuable resource for further validation.

## Supplementary data

Supplementary data are available at JXB online.

Text S1. Variation of anthocyanin content and composition in Syrah, Pinot Noir, and their progeny.

Figure S1. Variation of anthocyanin content in the Syrah×Pinot Noir progeny.

Figure S2. Validation of the pseudoreplicates approach in the gene expression study.

Figure S3. Evolutionary relationships of 108 BAHD proteins from grapevine and other plant species.

Table S1. Main features of the Syrah×Pinot Noir map.

Table S2. Anthocyanin profile of the parental varieties.

Table S3. Anthocyanin variation in the coloured individuals of the Syrah×Pinot Noir progeny.

Table S4. Correlations between metabolites in the coloured progeny.

Table S5. List of QTLs for anthocyanin content and composition identified in the Syrah×Pinot Noir progeny.

Table S6. List of the GrapeGen Chip probe sets differentially expressed during development in high and low anthocyanin producers.

Table S7. Test for over-representation of specific functional categories in QTL regions.

Supplementary Data
