# Supplementary material for: New candidate genes for the fine regulation of the colour of grapes
Source: J Exp Bot. 2015 Jun 12;66(15):4427–40. doi: 10.1093/jxb/erv159 (PMC4507754; doi:10.1093/jxb/erv159)
Supplement: Supplementary Data [file supp_66_15_4427__index.html]

New candidate genes for the fine regulation of the colour of grapes — New candidate genes for the fine regulation of the colour of grapes — Supplementary Data 

# New candidate genes for the fine regulation of the colour of grapes

## Supplementary Data

Data files

**Files in this Data Supplement:**

- Supplementary Data - Supplementary Data
- Supplementary Data - Supplementary Data
