# Supplementary material for: New candidate genes for the fine regulation of the colour of grapes
Source: J Exp Bot. 2015 Jun 12;66(15):4427–40. doi: 10.1093/jxb/erv159 (PMC4507754; doi:10.1093/jxb/erv159)
Supplement: Supplementary Data [file supp_erv159_jexbot144097_file002.pdf]

Journal of Experimental Botany

New candidate genes for the fine regulation of grapes' color

Costantini Laura, Malacarne Giulia, Lorenzi Silvia, Troggio Michela, Mattivi Fulvio, Moser Claudio, Grando Maria Stella

SUPPLEMENTARY DATA

Text S1. Variation of anthocyanin content and composition in Syrah, Pinot Noir and their progeny.

Syrah and Pinot Noir accumulated variable amounts of anthocyanins in the four seasons, ranging from 274 to 1021 mg/kg for Syrah and from 295 to 587 mg/kg for Pinot Noir. Nevertheless, they showed a relatively stable profile, with the following conserved features: Pinot Noir didn't accumulate any acylated form, whereas Syrah accumulated on average 19% of anthocyanins in their acetate form and 33% of anthocyanins in their *p*-coumarate form; the absolute amount of anthocyanins in Pinot Noir was more or less half of that in Syrah (with the exception of 2009); in both cultivars malvidin 3-monoglucoside and peonidin 3-monoglucoside (this last especially in Pinot Noir) were the most abundant metabolites; the ratio between tri-hydroxylated and di-hydroxylated anthocyanins (triOH/diOH) was on average 2 times higher in Syrah, whereas the ratio between 3'methylated and 3'hydroxylated anthocyanins (3'Meth/3'OH) and the ratio between 3'5'methylated and 3'5'hydroxylated anthocyanins (3'5'Meth/3'5'OH) were on average 3 and 4 times higher in Pinot Noir, respectively (Table S2).

The F_1 progeny included approximately 25% of individuals with white berries, in agreement with a 1:3 segregation of a major locus and with a bi-modal distribution of white-to-colored grapes for anthocyanin content (Figure 1 and Figure S1). The colored offspring showed extensive variation in the content of all the investigated compounds, with total anthocyanins ranging from 37 to 2391 mg/kg of berry, and a transgressive segregation for the five monoglucosides, their sum and the total anthocyanins (Table S3 and Figure S1). As for the acetate and *p*-coumarate forms of the five anthocyanins and their sums, the transgression concerned the maximum values, whereas the minimum values (zero) were detected only in Pinot Noir, meaning that none of the progeny individuals inherited the total lack of esters from their male parent.

Trait distribution was significantly (p-value < 0.05) different from a normal curve when tested on the whole progeny, due to the presence of the zero class. When analyzing the set of colored individuals, most of the traits (peonidin and malvidin among the metabolites expressed as absolute amounts, and almost all the ratios) were distributed according to a normal curve (data not shown).

Spearman rank-order correlations between years were significant at the 0.01 level for all the metabolites and groups of metabolites (Figure 1 and Figure S1). R values were high, confirming that anthocyanin biosynthesis is not strongly affected by environmental conditions, and ranged from

0.79 (for cyanidin 3-monoglucoside-acetate) to 0.95 (for delphinidin and petunidin 3-monoglucoside-*p*-coumarate) when analyzing the whole progeny and from 0.53 (for cyanidin 3-monoglucoside-*p*-coumarate) to 0.85 (for delphinidin 3-monoglucoside-*p*-coumarate) when analyzing only the colored progeny.

Spearman rank-order correlations between metabolites were also significant at the 0.01 level (Table S4), consistent with the fifteen anthocyanins being synthesized through the same biosynthetic pathway. However, further analysis of their correlation coefficients may suggest traits which are controlled by the same gene(s), e.g. the same methyltransferase for the highly correlated 3'Meth/3'OH and 3'5'Meth/3'5'OH ratios (r = 0.85, Table S4A) or *viceversa* traits with a peculiar determination and, consequently, reaction steps for which metabolic QTLs may be found, as in the case of di-hydroxylated anthocyanins (especially peonidin) in respect to tri-hydroxylated ones (r ranging from 0.43 to 0.46 for the correlation between peonidin derivatives and the derivatives of tri-hydroxylated anthocyanins, Table S4A).

Supplementary **Table S1.** Main features of the Syrah x Pinot Noir linkage map.

Abbreviations: N = number; Min = minimum; Max = maximum; SSR = simple sequence repeat; BES = BAC end sequence; eSNP = electronic single nucleotide polymorphism; cM = centiMorgan; LG = linkage group.

SSR markers belonged to the following series: UDV (Di Gaspero *et al.*, 2005), VMC (Vitis Microsatellite Consortium), VrZAG (Sefc *et al.*, 1999), VVI (Merdinoglu *et al.*, 2005) and VVMD (Bowers *et al.*, 1996 and 1999).

	N	Average	Min	Max
**N. of scored markers**	690			
**Marker type**				
Morphological	1			
SSR	49			
BES	7			
eSNP	633			
**Segregation type**				
abxcd	15			
efxeg	22			
hkxhk	263			
lmxll	10			
nnxnp	380			
**N. of discarded markers**	36			
**N. of mapped markers**	593			
**N. of markers per linkage group**		31	18 (LG14)	46 (LG18)
**Distance between markers (cM)**		2	1 (LG9)	3 (LG17)
**N. of gaps 10-20 cM**	16			
**N. of gaps 10-20 cM per linkage group**			0	3 (LGs 7, 8)
**N. of gaps > 20 cM**	3			
**N. of gaps > 20 cM per linkage group**			0	1 (LGs 1, 4, 11)

References

Bowers JE, Dangl GS, Meredith CP. 1999. Development and characterization of additional microsatellite DNA markers for grape. *American Journal of Enology and Viticulture* **50**, 243–246.

Bowers JE, Dangl GS, Vignani R, Meredith CP. 1996. Isolation and characterization of the new polymorphic simple sequence repeat loci in grape (*Vitis vinifera* L.). *Genome* **45**, 1142–1149.

Di Gaspero G, Cipriani G, Marrazzo MT, Andreetta D, Prado Castro MJ, Peterlunger E, Testolin R. 2005. Isolation of (AC)*n*-microsatellites in *Vitis vinifera* L. and analysis of genetic background in grapevines under marker assisted selection. *Molecular Breeding* **15**, 11–20.
Merdinoglu D, Butterlin G, Bevilacqua L, Chiquet V, Adam-Blondon AF, Decroocq S. 2005. Development and characterization of a large set of microsatellite markers in grapevine (*Vitis vinifera* L.) suitable for multiplex PCR. *Molecular Breeding* **15**, 349–366.
Sefc KM, Regner F, Turetschek E, Glössl J, Steinkellner H. 1999 Identification of microsatellite sequences in *Vitis riparia* and their applicability for genotyping of different Vitis species. *Genome* **42**, 367–373.

Table S2. Anthocyanin profile (percentage), total concentration of anthocyanins (mg/kg) and some ratios concerning hydroxylation and methylation observed in the parental varieties in four seasons.

Abbreviations: Cya3M = cyanidin 3-monoglucoside; Peo3M = peonidin 3-monoglucoside; Del3M = delphinidin 3-monoglucoside; Pet3M = petunidin 3-monoglucoside; Mal3M = malvidin 3-monoglucoside; triOH/diOH = (delphinidin + petunidin + malvidin 3-monoglucoside)/(cyanidin + peonidin 3-monoglucoside); 3'Meth/3'OH = peonidin 3-monoglucoside/cyanidin 3-monoglucoside; 3'5'Meth/3'5'OH = malvidin 3-monoglucoside/delphinidin 3-monoglucoside.

		%							Total anthocyanins	ratio		
		**Cya3M**	**Peo3M**	**Del3M**	**Pet3M**	**Mal3M**	**Sum of acetates**	**Sum of *p*-coumarates**		**triOH/ diOH**	**3'Meth/ 3'OH**	**3'5'Meth/ 3'5'OH**
**2007**	**Syrah**	1.26	7.89	6.77	7.01	34.59	17.97	24.52	1021.35	5.29	6.26	5.11
	**Pinot Noir**	1.65	26.53	2.46	3.98	65.38	0.00	0.00	587.39	2.55	16.05	26.58
**2008**	**Syrah**	0.96	6.41	6.18	6.46	32.13	19.15	28.71	827.56	6.07	6.67	5.20
	**Pinot Noir**	1.08	18.93	2.97	4.65	72.29	0.00	0.00	431.98	3.99	17.51	24.36
**2009**	**Syrah**	0.37	5.16	1.77	3.18	26.57	20.64	42.30	273.73	5.70	13.98	15.00
	**Pinot Noir**	0.90	27.00	1.38	3.11	67.28	0.00	0.00	298.47	2.57	30.07	48.62
**2011**	**Syrah**	1.09	8.32	3.72	4.81	27.01	16.91	38.13	470.09	3.78	7.64	7.25
	**Pinot Noir**	0.97	29.16	2.49	3.99	63.38	0.00	0.00	295.49	2.32	29.92	25.41
**Average**	**Syrah**	0.92	6.95	4.61	5.37	30.08	18.67	33.41	648.18	5.21	8.64	8.14
	**Pinot Noir**	1.15	25.40	2.33	3.93	67.08	0.00	0.00	403.33	2.86	23.38	31.24

Supplementary Table S3. Range of variation of each anthocyanin or group of anthocyanins (expressed as mg/kg of berries) in the colored individuals of the Syrah x Pinot Noir progeny in four seasons. Minimum values were considered different from zero when they exceeded malvidin 3-monoglucoside limit of quantification that is 0.45 mg/kg.

Abbreviations: Cya3M = cyanidin 3-monoglucoside; Peo3M = peonidin 3-monoglucoside; Del3M = delphinidin 3-monoglucoside; Pet3M = petunidin 3-monoglucoside; Mal3M = malvidin 3-monoglucoside; Ac = acetate; Cou = *p*-coumarate; Tot3M = sum of anthocyanins in their 3-monoglucoside form; Tot3MAc = sum of anthocyanins in their acetate form; Tot3MCou = sum of anthocyanins in their *p*-coumarate form; Tot_Anth = total anthocyanins; Min = minimum; Max = maximum.

**Metabolite**	**2007**		**2008**		**2009**		**2011**	
	**Min**	**Max**	**Min**	**Max**	**Min**	**Max**	**Min**	**Max**
Cya3M	0.50	40.36	0.53	43.54	0.00	22.36	0.56	26.80
Peo3M	8.29	208.93	9.91	190.12	0.00	125.57	3.85	159.99
Del3M	1.99	365.71	4.13	278.93	0.53	147.81	4.30	172.58
Pet3M	3.53	319.94	3.07	230.29	0.00	140.45	6.14	161.08
Mal3M	90.02	1033.55	119.89	1001.82	17.07	778.32	34.01	571.27
Cya3MAc	0.00	5.23	0.00	6.67	0.00	3.84	0.00	4.24
Peo3MAc	2.52	35.47	2.18	41.96	0.80	31.20	3.21	30.87
Del3MAc	0.56	70.61	0.75	55.69	0.00	40.24	0.00	46.25
Pet3MAc	0.00	76.87	0.69	55.50	0.00	43.34	0.52	43.61
Mal3MAc	19.91	359.20	3.65	291.87	2.25	211.77	18.17	185.91
Cia3MCou	0.00	15.82	0.00	10.33	0.00	9.05	0.91	9.59
Peo3MCou	0.68	71.05	4.16	54.75	1.31	43.27	6.02	62.20
Del3MCou	0.00	42.28	0.56	32.20	0.00	22.42	0.80	36.55
Pet3MCou	0.76	39.44	1.14	33.88	0.00	24.05	1.30	31.19
Mal3MCou	27.29	226.65	22.92	317.00	3.92	238.66	25.42	196.11
Tot3M	108.68	1683.19	176.25	1560.04	27.60	1100.94	155.62	984.02
Tot3MAc	24.59	534.83	18.35	382.77	3.40	312.31	32.70	299.45
Tot3MCou	34.78	322.41	34.46	400.51	6.48	301.17	60.21	294.21
Tot_Anth	168.05	2391.49	280.71	2173.07	37.48	1640.54	263.82	1545.60

Supplementary **Table S4.** Spearman rank-order correlations between metabolites in the colored progeny. Correlations coefficients are average values from four seasons (the range of variation is reported in brackets).

Abbreviations: Cya3M = cyanidin 3-monoglucoside; Peo3M = peonidin 3-monoglucoside; Del3M = delphinidin 3-monoglucoside; Pet3M = petunidin 3-monoglucoside; Mal3M = malvidin 3-monoglucoside; Ac = acetate; Cou = *p*-coumarate; Acylated = 3-monoglucoside-acetate + 3-monoglucoside-*p*-coumarate; Der = derivatives (3-monoglucoside + 3-monoglucoside-acetate + 3-monoglucoside-*p*-coumarate); diOH = di-hydroxylated (cyanidin + peonidin 3-monoglucoside); triOH = tri-hydroxylated (delphinidin + petunidin + malvidin 3-monoglucoside); 3’Meth/3’OH = peonidin 3-monoglucoside/cyanidin 3-monoglucoside; 3’5’Meth/3’5’OH = malvidin 3-monoglucoside/delphinidin 3-monoglucoside.

A)	**CyaDer**	**PeoDer**	**DelDer**	**PetDer**	**diOH**	**3’Meth/ 3’OH**
**PeoDer**	0.75 (0.71/0.81)					
**DelDer**	0.79 (0.73/0.84)	0.46 (0.39/0.50)				
**PetDer**	0.77 (0.70/0.83)	0.46 (0.38/0.50)	0.98 (0.97/0.99)			
**MalDer**	0.53 (0.48/0.59)	0.43 (0.37/0.48)	0.77 (0.74/0.78)	0.81 (0.79/0.83)		
**triOH**					0.49 (0.42/0.55)	
**3’5’Meth/ 3’5’OH**						0.85 (0.75/0.91)

B)	**Acylated cyanidin**	**Acylated peonidin**	**Acylated delphinidin**	**Acylated petunidin**	**Acylated malvidin**
**Cya3M**	0.63 (0.35/0.85)				
**Peo3M**		0.86 (0.84/0.88)			
**Del3M**			0.94 (0.91/0.96)		
**Pet3M**				0.93 (0.90/0.96)	
**Mal3M**					0.83 (0.71/0.90)

C)	**Cya3MCou**	**Peo3MCou**	**Del3MCou**	**Pet3MCou**	**Mal3MCou**
**Cya3MAc**	0.59 (0.29/0.81)				
**Peo3MAc**		0.83 (0.80/0.86)			
**Del3MAc**			0.92 (0.91/0.92)		
**Pet3MAc**				0.88 (0.86/0.89)	
**Mal3MAc**					0.83 (0.80/0.88)

Correlations are significant at the 0.01 level

Supplementary **Figure S1.** Variation of anthocyanin content in the Syrah x Pinot Noir progeny in four different vintages. For the two parents averaged values across years are reported. In the right upper part of each plot the Spearman rank-order correlation between years (mean value and range of variation in brackets) is shown for the whole progeny (top) and the colored progeny (bottom). Correlations are significant at the 0.01 level.

Abbreviations: Sy = Syrah; PN = Pinot Noir; conc = concentration.

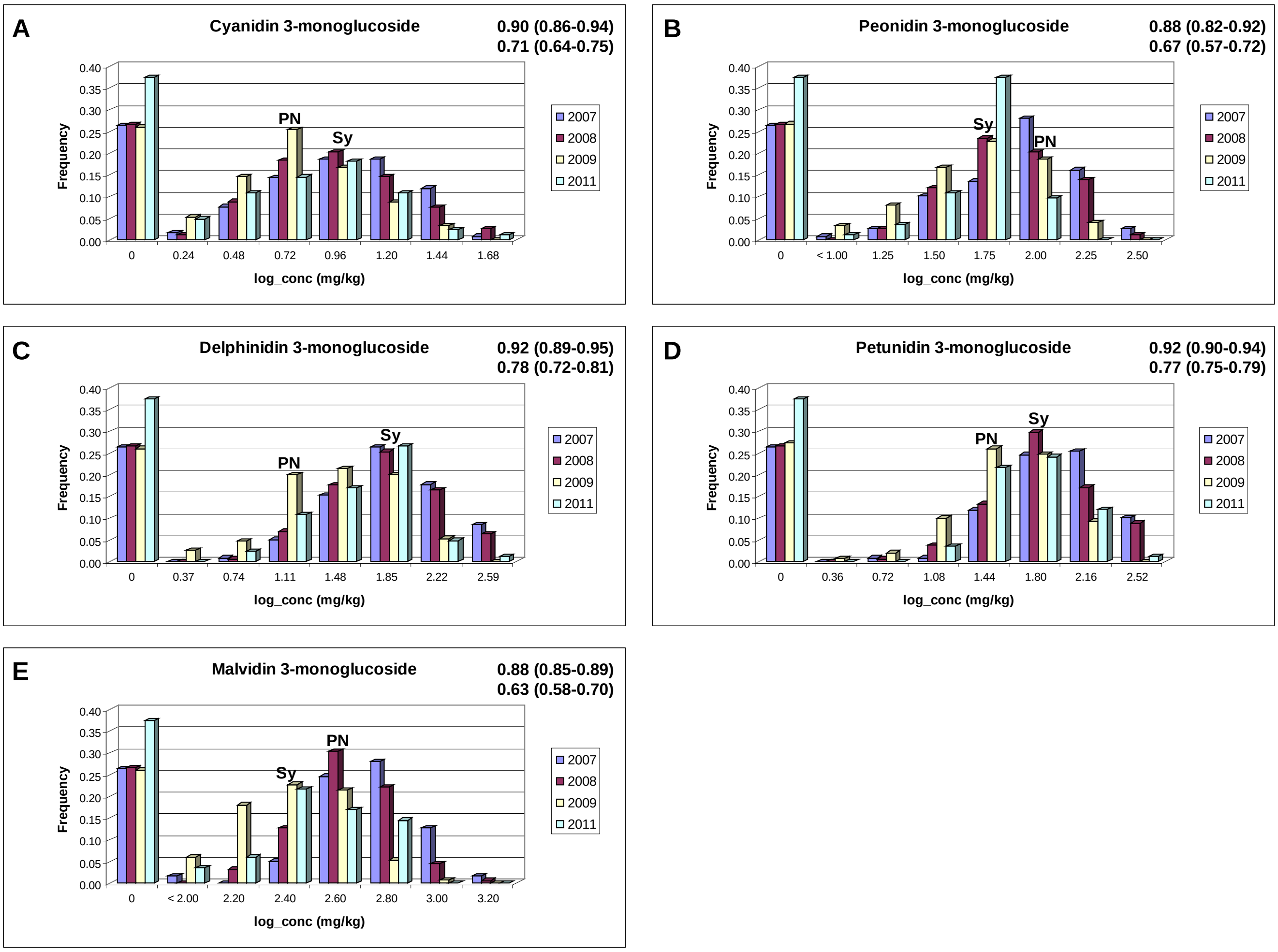

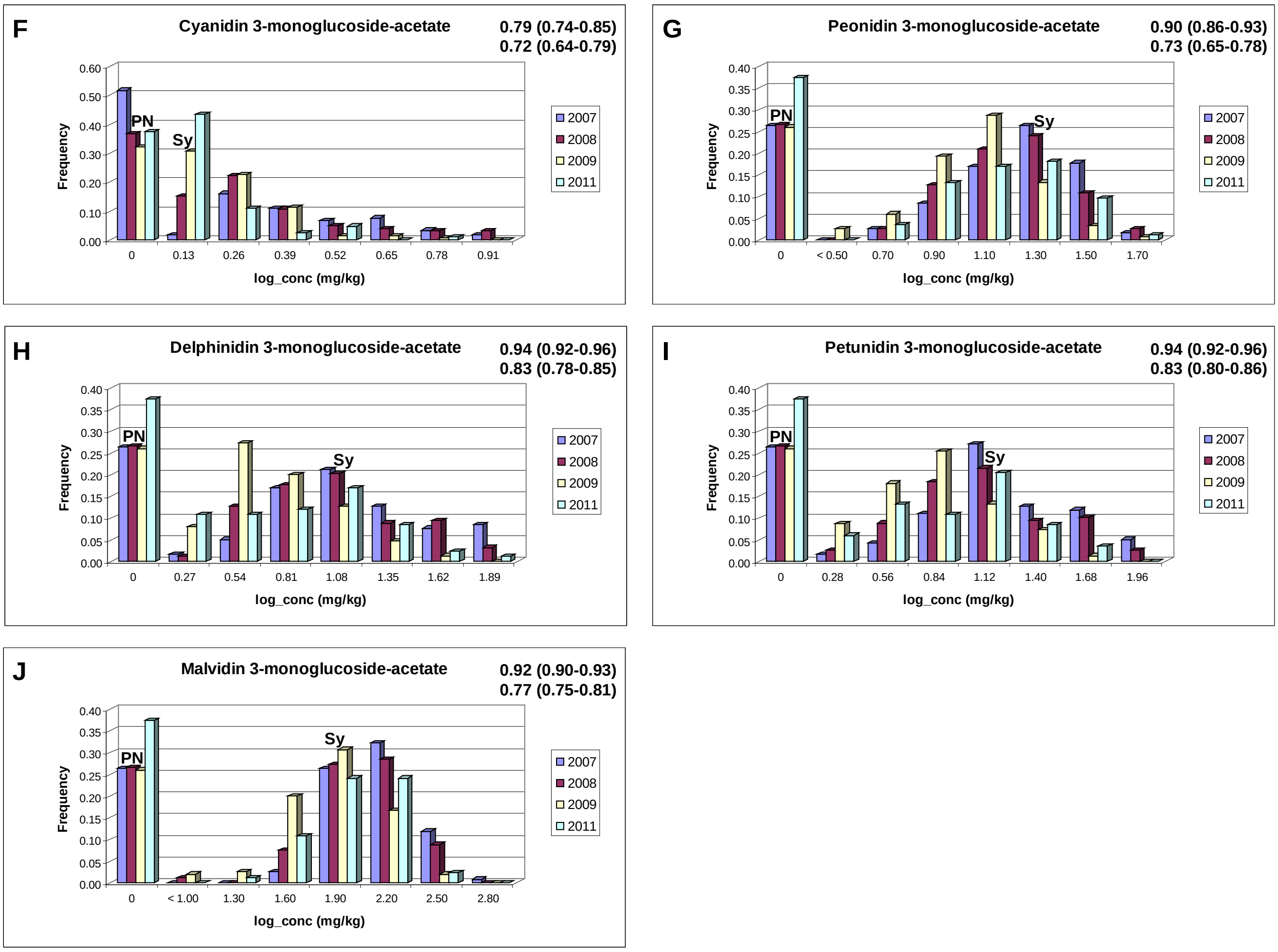
F
Cyanidin 3-monoglucoside-acetate
0.79 (0.74-0.85)
0.72 (0.64-0.79)
Frequency
PN
Sy
log_conc (mg/kg)
0 0.13 0.26 0.39 0.52 0.65 0.78 0.91
2007 2008 2009 2011
G
Peonidin 3-monoglucoside-acetate
0.90 (0.86-0.93)
0.73 (0.65-0.78)
Frequency
PN
Sy
log_conc (mg/kg)
0 < 0.50 0.70 0.90 1.10 1.30 1.50 1.70
2007 2008 2009 2011
H
Delphinidin 3-monoglucoside-acetate
0.94 (0.92-0.96)
0.83 (0.78-0.85)
Frequency
PN
Sy
log_conc (mg/kg)
0 0.27 0.54 0.81 1.08 1.35 1.62 1.89
2007 2008 2009 2011
I
Petunidin 3-monoglucoside-acetate
0.94 (0.92-0.96)
0.83 (0.80-0.86)
Frequency
PN
Sy
log_conc (mg/kg)
0 0.28 0.56 0.84 1.12 1.40 1.68 1.96
2007 2008 2009 2011
J
Malvidin 3-monoglucoside-acetate
0.92 (0.90-0.93)
0.77 (0.75-0.81)
Frequency
PN
Sy
log_conc (mg/kg)
0 < 1.00 1.30 1.60 1.90 2.20 2.50 2.80
2007 2008 2009 2011

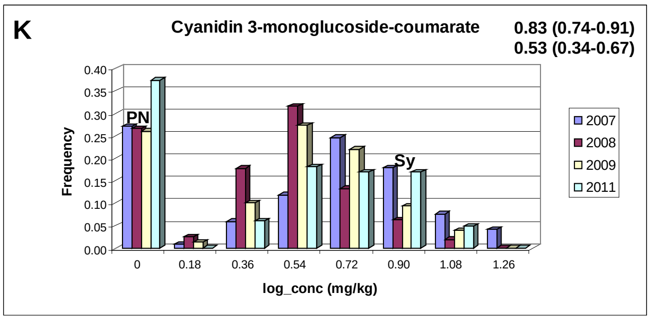
K
Cyanidin 3-monoglucoside-coumarate
0.83 (0.74-0.91)
0.53 (0.34-0.67)
Frequency
0.40
0.35
0.30
0.25
0.20
0.15
0.10
0.05
0.00
PN
Sy
0
0.18
0.36
0.54
0.72
0.90
1.08
1.26
log_conc (mg/kg)
2007
2008
2009
2011

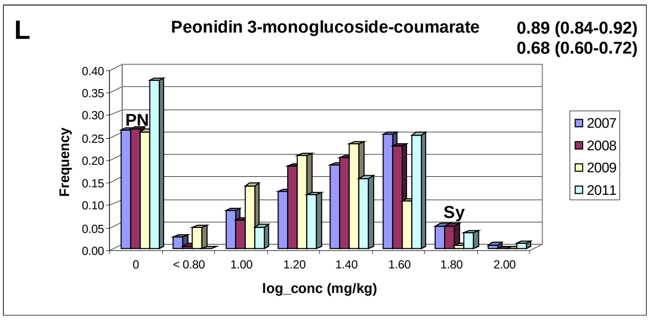
L
Peonidin 3-monoglucoside-coumarate
0.89 (0.84-0.92)
0.68 (0.60-0.72)
Frequency
0.40
0.35
0.30
0.25
0.20
0.15
0.10
0.05
0.00
PN
Sy
0
< 0.80
1.00
1.20
1.40
1.60
1.80
2.00
log_conc (mg/kg)
2007
2008
2009
2011

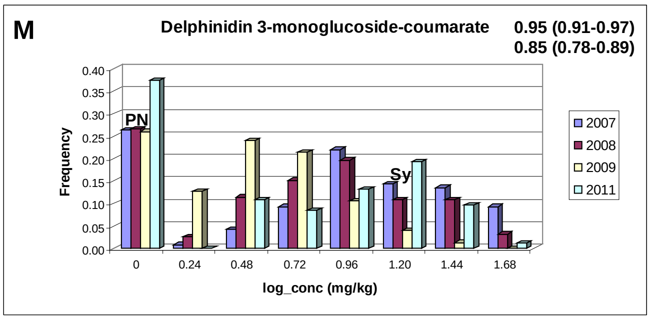
M
Delphinidin 3-monoglucoside-coumarate
0.95 (0.91-0.97)
0.85 (0.78-0.89)
Frequency
0.40
0.35
0.30
0.25
0.20
0.15
0.10
0.05
0.00
PN
Sy
0
0.24
0.48
0.72
0.96
1.20
1.44
1.68
log_conc (mg/kg)
2007
2008
2009
2011

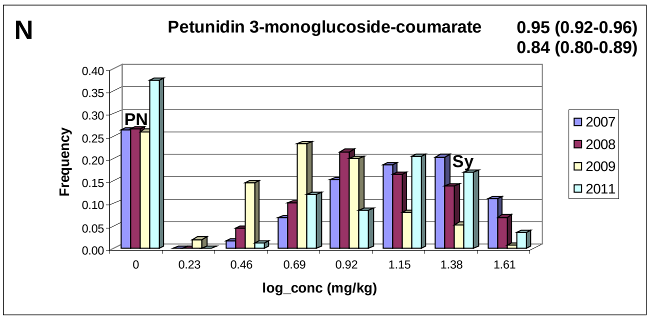
N
Petunidin 3-monoglucoside-coumarate
0.95 (0.92-0.96)
0.84 (0.80-0.89)
Frequency
0.40
0.35
0.30
0.25
0.20
0.15
0.10
0.05
0.00
PN
Sy
0
0.23
0.46
0.69
0.92
1.15
1.38
1.61
log_conc (mg/kg)
2007
2008
2009
2011

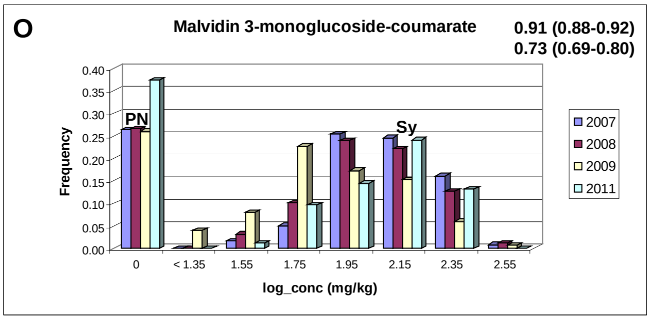
O
Malvidin 3-monoglucoside-coumarate
0.91 (0.88-0.92)
0.73 (0.69-0.80)
Frequency
0.40
0.35
0.30
0.25
0.20
0.15
0.10
0.05
0.00
PN
Sy
0
< 1.35
1.55
1.75
1.95
2.15
2.35
2.55
log_conc (mg/kg)
2007
2008
2009
2011

Supplementary **Figure S2.** Consistency of expression tested among experiments (A) and replicates (B) for *VvMYBA1* and *VvUFGT* (*UDP-glucose flavonoid 3-O-glucosyltransferase*) genes.

A) Blue lines represent the expression levels as assessed by microarray analysis and reported as means and standard errors of the three genotypes (pseudoreplicates) selected as low- and high-anthocyanin producers at three developmental stages in 2007. Histograms represent the relative expression levels (fold-change relative to the average expression value of all the genotypes in all the stages) as assessed by real-time RT-PCR and reported as means and standard errors of the three genotypes (pseudoreplicates) at the three stages. The following primers were used for real-time RT-PCR: VvMYBA1_For = 5'-TAGTCACCACTTCAAAAAGG-3', VvMYBA1_Rev = GAATGTGTTTGGGGTTTATC; VvUFGT_For = 5'- GGGATGGTAATGGCTGTGG-3', VvUFGT_Rev = 5'-ACATGGGTGGAGAGAGTGAGTT-3'. The two constitutive genes *GAPDH* and *ACTIN* (Reid *et al.*, 2006) were employed for normalization.

B) Histograms represent the expression levels as assessed by real-time RT-PCR and reported as means and standard errors of the three genotypes (pseudoreplicates) selected as low- (white bar) and high- (black bar) anthocyanin producers at three developmental stages in 2007, and as means and standard errors of three biological replicates for each genotype at the three stages in 2011 (bar with light grey grid = LAPs, bar with black grid = HAPs). Genotype codes are shown in the legend. Asterisks indicate significant changes ($p<0.05$) in the comparison between LAPs and HAPs at each stage in each season, tested by a Student's t test performed on the log 2 transformed data.

Although the samples for the analysis were collected in two different seasons, the range of variability calculated as standard error among different genotypes (samples collected in 2007) and among biological replicates of the same genotype (samples collected in 2011) proved to be comparable, or even higher in the second case. In particular, it is evident that the two groups of genotypes (LAPs and HAPs) are significantly different in the expression level of *VvMYBA1* and *VvUFGT*, encoding for the known regulator and enzyme involved in the biosynthesis of anthocyanins, in agreement with the different anthocyanin level at maturity.

Abbreviations: RMA = Robust Multi-array Average; NRQ = Normalized Relative Quantity; PV = pre-véraison; VER = véraison; MAT = maturity, LAPs = low anthocyanin producers, HAPs = high anthocyanin producers, 3BR = three biological replicates.

Reference

Reid KE, Olsson N, Schlosser J, Peng F, Lund ST. 2006. An optimized grapevine RNA isolation procedure and statistical determination of reference genes for real-time RT-PCR during berry development. *BMC Plant Biology* **6**, 27.

A

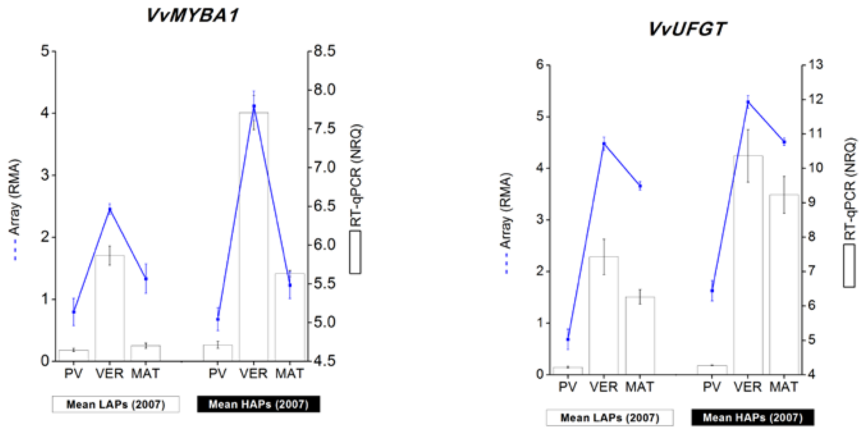
VvMYBA1
Array (RMA)
RT-qPCR (NRQ)
PV VER MAT PV VER MAT
Mean LAPs (2007)
Mean HAPs (2007)
VvUFGT
Array (RMA)
RT-qPCR (NRQ)
PV VER MAT PV VER MAT
Mean LAPs (2007)
Mean HAPs (2007)

B

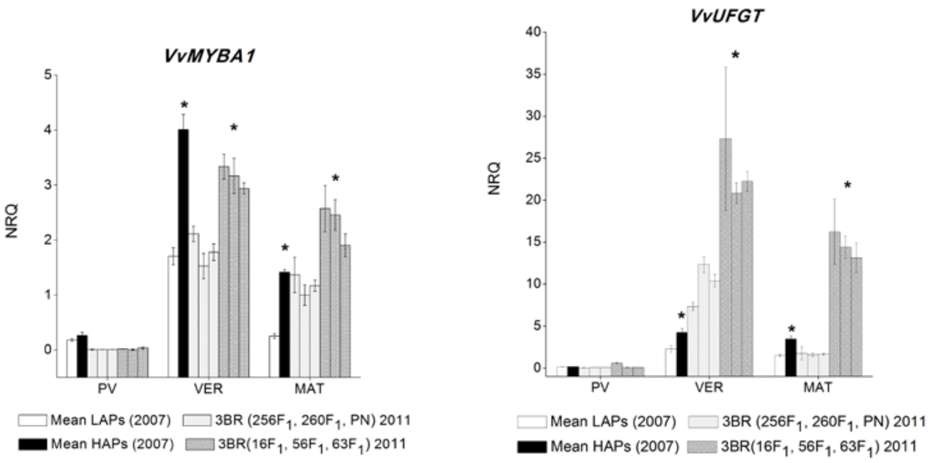
VvMYBA1
NRQ
PV
VER
MAT
Mean LAPs (2007)
3BR (256F1, 260F1, PN) 2011
Mean HAPs (2007)
3BR(16F1, 56F1, 63F1) 2011
VvUFGT
NRQ
PV
VER
MAT
Mean LAPs (2007)
3BR (256F1, 260F1, PN) 2011
Mean HAPs (2007)
3BR(16F1, 56F1, 63F1) 2011

Supplementary **Figure S3.**

A) Exon-intron structure of the nine BAHD acyltransferases underlying the QTL on LG 12b, drawn with the online tool FancyGene (Rambaldi and Ciccarelli, 2009). Arrows indicate the DNA strand; thin and thick boxes correspond to untranslated regions (UTRs) and exons, respectively; lines represent introns (in scale, except for intron in VvAT6, which is truncated for better visualization); colored boxes are conserved motifs.

VvAT1 = VIT_12s0134g00580; VvAT2 = VIT_12s0134g00590; VvAT3 = VIT_12s0134g00600; VvAT4 = VIT_12s0134g00620; VvAT5 = VIT_12s0134g00630; VvAT6 = VIT_12s0134g00640; VvAT7 = VIT_12s0134g00650; VvAT8 = VIT_12s0134g00660; VvAT9 = VIT_12s0134g00670).

B) Evolutionary relationships of 108 BAHD proteins from grapevine and other plant species reconstructed in MEGA5 (Hall *et al.*, 2013). An initial number of 126 protein sequences were scored: the 9 grapevine putative acyltransferases (VvAT1-VvAT9) found in the confidence interval of the QTL on LG 12b, additional 53 grapevine putative BAHD acyltransferases and 64 biochemically characterized plant BAHD acyltransferases acquired from the work of Tuominen *et al.* (2011). Sequence alignment was first conducted with Muscle (Edgar, 2004), then evaluated through the web-based program Guidance (http://guidance.tau.ac.il/) and manually curated in BioEdit 7.2.0 (Hall *et al.*, 1999). The aligned sequences were screened for the presence of two highly conserved motifs among BAHD acyltransferases (HXXXD and DFGWG-like) and removed from subsequent analysis when either motif was absent (with the exception of Clade II members). VvAT1-9 proteins were additionally screened for the presence of specific signatures of anthocyanin acyltransferases (LTFFD and YFGNC) (Tuominen *et al.*, 2011). After this screening, two VvATs (VvAT1 and VvAT9) and 106 additional BAHD sequences were retained for phylogenetic analysis. The phylogenetic tree was constructed according to the maximum likelihood method by using the WAG model coupled with five discrete categories of a gamma distribution to account for among-site rate variations, invariant sites and an empirical estimation of the amino acid frequencies. The accuracy of this reconstruction was assessed through bootstrap with 100 replicates (Felsenstein, 1985). All positions containing alignment gaps and missing data were eliminated only in pairwise sequence comparisons (Pairwise deletion option). Bootstrap values lower than 40% are not shown.

Accession numbers of the proteins reported in the tree are as follows:

Clade Ia: Ss3AT (hydroxycinnamoyl-CoA:anthocyanin 3-O-glucoside-6"-O-hydroxycinnamoyltransferase from *Salvia splendens*, AAR28757.1), Pf3AT (hydroxycinnamoyl-CoA:anthocyanin 3-O-glucoside-6''-O-acyltransferase from *Perilla frutescens*, BAA93475.1), Ss5MaT1 (malonyl-CoA:anthocyanin 5-O-glucoside-6'''-O-malonyltransferase from *Salvia splendens*, AAL50566.1), Pf5MaT (malonyl-CoA:anthocyanin 5-O-glucoside-6'''-O-

malonyltransferase from *Perilla frutescens*, AAL50565.1), Vh3MaT1 (quercetin 3-O-glucoside-6''-O-malonyltransferase from *Verbena hybrida*, AAS77402.1), Lp3MaT1 (quercetin 3-O-glucoside-6''-O-malonyltransferase from *Lamium purpureum*, AAS77404.1), NtMaT1 (malonyl-CoA flavonoid/napthol glucoside acyltransferase from *Nicotiana tabacum*, BAD93691.1), Gt5AT (hydroxycinnamoyl-CoA:anthocyanin 5-O-glucoside-6'''-O-acyltransferase from *Gentiana triflora*, BAA74428.1), VvAT9 (anthocyanin 5-aromatic acyltransferase from *Vitis vinifera*, VIT_12s0134g00670), VvAT1 (hydroxycinnamoyl-CoA quinate hydroxycinnamoyltransferase from *Vitis vinifera*, VIT_12s0134g00580), Dv3MaT (malonyl-CoA:anthocyanidin 3-O-glucoside-6''-O-malonyltransferase from *Dahlia variabilis*, AAO12206.1), Sc3MaT (malonyl-CoA:anthocyanidin 3-O-glucoside-6''-O-malonyltransferase from *Senecio cruentus*, AAO38058.1), Dm3MaT2 (anthocyanidin 3-O-glucoside-3'',6''-O-dimalonyltransferase from *Dendranthema x morifolium*, AAQ63616.1), Dm3MaT1 (malonyl-CoA:anthocyanidin 3-O-glucoside-6''-O-malonyltransferase from *Dendranthema x morifolium*, AAQ63615.1), At3AT2 (coumaroyl-CoA:anthocyanidin 3-O-glucoside-6''-O-coumaroyltransferase 2 from *Arabidopsis thaliana*, NP_171849.2), At3AT1 (coumaroyl-CoA:anthocyanidin 3-O-glucoside-6''-O-coumaroyltransferase 1 from *Arabidopsis thaliana*, NP_171890.1), At5MAT (malonyl-CoA:anthocyanidin 5-O-glucoside-6''-O-malonyltransferase from *Arabidopsis thaliana*, NP_189600.1)

Clade Ib: VIT_16s0050g00870 (acyltransferase), VIT_16s0050g00830 (beta-ketoacyl-CoA synthase), VIT_07s0031g00270 (anthranilate N-hydroxycinnamoyl/benzoyltransferase), VIT_07s0031g00280 (anthranilate N-hydroxycinnamoyl/benzoyltransferase), VIT_18s0001g10180 (N-hydroxycinnamoyl/benzoyltransferase 3), VIT_08s0007g01930 (anthranilate N-benzoyltransferase), VIT_13s0019g03430 (anthocyanin 5-aromatic acyltransferase)

Clade IIIa: FvVAAT (alcohol acyltransferase from *Fragraria vesca*, CAC09062.1), FaSAAT (alcohol acyltransferase from *Fragaria x ananassa*, AAG13130.1), RhAAT1 (acetyl-CoA geraniol/citronellol acetyltransferase from *Rosa* hybris cultivar, AAW31948.1), VIT_18s0001g00450 (vinorine synthase), CmAAT4 (putative alcohol acyltransferase from *Cucumis melo*, AAW51126.1), RsVISY (vinorine synthase from *Rauvolfia serpentine*, CAD89104.2), CbBEAT (acetyl-CoA: benzylalcohol acetyltransferase from *Clarkia breweri*, AAC18062.1), PsSalAT (salutaridinol 7-O-acetyltransferase from *Papaver somniferum*, AAK73661.1), Ss5MaT2 (malonyl-CoA:anthocyanin 5-O-glucoside-4'''-O-malonyltransferase from *Salvia splendens*, AAR26385.1), CaPun1 (acyltransferase from *Capsicum annum*, AAV66311.1), CrMAT (minovincinine 19-hydroxy-O-acetyltransferase from *Catharanthus roseus*,

AAO13736.1), CrDAT (deacetylvindoline 4-O-acetyltransferase from *Catharanthus roseus*, AAC99311.1)

Clade IIIb: VIT_09s0096g00680 (C2-HC type zinc finger protein C.e-MyT1), VIT_09s0096g00660 (C2-HC type zinc finger protein C.e-MyT1), VIT_09s0096g00430 (C2-HC type zinc finger protein C.e-MyT1), VIT_09s0054g01730 (coniferyl alcohol acyltransferase), VIT_09s0096g00510 (C2-HC type zinc finger protein C.e-MyT1), VIT_09s0096g00550 (C2-HC type zinc finger protein C.e-MyT1), VIT_09s0054g01740 (C2-HC type zinc finger protein C.e-MyT1), VIT_09s0054g01700 (C2-HC type zinc finger protein C.e-MyT1), PhCFAT (coniferyl alcohol acyltransferase from *Petunia x hybrida*, ABG75942.1), VIT_02s0087g00370 (anthranilate N-hydroxycinnamoyl/benzoyltransferase)

Clade II: VIT_14s0030g01950 (transferase), VIT_04s0008g04800 (transferase), AtCER2 from *Arabidopsis thaliana* (AAM64817.1), ZmGlossy2 from *Zea mays* (CAA61258.1), VIT_05s0029g00480 (Eceriferum 2, CER2), VIT_18s0001g07640 (Eceriferum 2, CER2)

Clade IV: HvACT (agmatine coumaroyltransferase from *Hordeum vulgare*, AAO73071.1)

Clade Vb: CcaHQT (hydroxycinnamoyl-CoA:quinate hydroxycinnamoyltransferase from *Cynara cardunculus* var. *altilis*, ABK79690.1), CcsHQT (hydroxycinnamoyl-CoA:quinate hydroxycinnamoyltransferase from *Cynara cardunculus* var. *scolymus*, ABK79689.1), SlHQT (hydroxycinnamoyl CoA quinate transferase from *Solanum lycopersicum*, CAE46933.1), NtHQT (hydroxycinnamoyl-CoA quinate transferase from *Nicotiana tabacum*, CAE46932.1), VIT_11s0037g00440 (hydroxycinnamoyl-CoA shikimate/quinate hydroxycinnamoyltransferase), AsHHT1 (hydroxycinnamoyl-CoA:hydroxyanthranilate N-hydroxycinnamoyltransferase from *Avena sativa*, BAC78633.1), NtHCT (hydroxycinnamoyl-CoA: shikimate/quinate hydroxycinnamoyltransferase from *Nicotiana tabacum*, CAD47830.1), PrHCT (hydroxycinnamoyl-CoA:shikimate hydroxycinnamoyltransferase from *Pinus radiate*, ABO52899.1), VIT_09s0018g01190 (anthranilate N-benzoyltransferase), AtHCT (hydroxycinnamoyl-CoA shikimate/quinate hydroxycinnamoyltransferase from *Arabidopsis thaliana*, NP_199704.1), TpHCT1A (shikimate O-hydroxycinnamoyltransferase from *Trifolium pratense*, ACI16630.1), TpHCT1B (shikimate O-hydroxycinnamoyl transferase from *Trifolium pratense*, ACI28534.1), SsHCT (hydroxycinnamoyl transferase from *Solenostemon scutellarioides*, CAK55166.1), DcHCBT (hydroxycinnamoyl/benzoyl-CoA:anthranilate N-hydroxycinnamoyl/benzoyltransferase from *Dianthus caryophyllus*, CAB06430.1), TpHCT2 (malate O-hydroxycinnamoyl transferase from *Trifolium pratense*, ACI16631.1), VIT_11s0037g00570 (anthranilate N-benzoyltransferase), VIT_11s0037g00580 (anthranilate N-hydroxycinnamoyl/benzoyltransferase), VIT_11s0103g00200

(anthranilate N-benzoyltransferase), AtSHT (spermidine hydroxycinnamoyl transferase from *Arabidopsis thaliana*, AEC06845.1)

Clade Va: VIT_00s0207g00010 (anthranilate N-benzoyltransferase protein 1), VIT_17s0000g00950 (hydroxycinnamoyl-CoA quinate hydroxycinnamoyltransferase), AtASFT (omega-hydroxypalmitate O-feruloyltransferase from *Arabidopsis thaliana*, Q94CD1.1), VIT_12s0057g00930 (hydroxycinnamoyl-CoA:shikimate hydroxycinnamoyltransferase), VIT_13s0067g01410 (anthranilate N-hydroxycinnamoyl/benzoyltransferase), VIT_18s0001g12990 (anthranilate N-benzoyltransferase protein 1), VIT_11s0016g04400 (transferase), TcDBBT (2-debenzoyl-7,13-diacetylbaccatin III-2-O-benzoyltransferase from *Taxus cuspidate*, AAG38049.1), TcBAPT (baccatin III O-phenylpropanoyltransferase from *Taxus cuspidate*, AAL92459.1), TcTAT (taxadienol acetyltransferase from *Taxus cuspidate*, AAF34254.1), TcDBAT (10-deacetylbaccatin III-10-O-acetyltransferase from *Taxus cuspidate*, AAF27621.1), TcDBNTBT (3'-N-debenzoyltaxol -2’-deoxytaxol N-benzoyltransferase from *Taxus Canadensis*, AAM75818.1), Ih3AT1 (acyltransferase from *Iris x hollandica*, BAE72676.1), VIT_07s0005g02670 (3-N-debenzoyl-2-deoxytaxol N-benzoyltransferase), MsAAT (alcohol acyl transferase from *Musa sapientum*, CAC09063.1), VIT_10s0003g00900 (anthranilate N-hydroxycinnamoyl/benzoyltransferase), VIT_06s0004g07650 (taxadien-5-alpha-ol-O-acetyltransferase), AtSDT (spermidine disinapoyl acyltransferase from *Arabidopsis thaliana*, NP_179932), VIT_14s0006g01460 (acetyl coa:(z)-3-hexen-1-ol acetyltransferase, CHAT), AtCHAT (acetyl-CoA: cis-3-hexen-1-ol acetyltransferase from *Arabidopsis thaliana*, AAN09797.1), LaHMT/HLT ((-)-13alpha-hydroxymultiflorine/(+)-13alpha- hydroxylupanine O-tigloyltransferase from *Lupinus albus*, BAD89275.1), PhBPBT (benzoyl-CoA:benzyl alcohol/phenylethanol benzoyltransferase from *Petunia x hybrida*, AAU06226.1), NtBEBT (benzoyl-CoA: benzyl alcohol benzoyltransferase from *Nicotiana tabacum*, AAN09798.1), CmAAT3 (putative alcohol acyltransferase from *Cucumis melo*, AAW51125.1), VIT_16s0039g00570 (10-deacetylbaccatin III 10-O-acetyltransferase), CbBEBT (benzoyl-CoA: benzyl alcohol benzoyltransferase from *Clarkia breweri*, AAN09796.1), CmAAT2 (putative alcohol acyltransferase from *Cucumis melo*, AAL77060.1), MdAAT2 (alcohol acyl transferase from *Malus domestica*, AAS79797.1), MdAAT1 (alcohol acyltransferase from *Malus domestica*, AAU14879.2), VIT_02s0087g00470 (anthraniloyal-CoA: methanol anthraniloyal transferase), VIT_02s0087g00490 (10-deacetylbaccatin III 10-O-acetyltransferase), VIT_09s0018g01490 (anthraniloyal-CoA: methanol anthraniloyal transferase), VlAMAT (anthraniloyal-CoA: methanol anthraniloyaltransferase from *Vitis labrusca*, AAW22989.1), VIT_02s0033g01060 (anthraniloyal-CoA: methanol anthraniloyal transferase),

VIT_02s0033g01000 (anthraniloyal-CoA: methanol anthraniloyal transferase), VIT_02s0033g01020 (anthraniloyal-CoA: methanol anthraniloyal transferase).

References

Edgar RC. 2004. MUSCLE: a multiple sequence alignment method with reduced time and space complexity. *BMC Bioinformatics* **5**, 113.

Felsenstein J. 1985. Confidence limits on phylogenies: an approach using the bootstrap. Evolution **39**, 783–791.

Hall BG. 2013. Building phylogenetic trees from molecular data with MEGA. *Molecular Biology and Evolution* **30**, 1229–1235.

Hall TA. 1999. BioEdit: a user-friendly biological sequence alignment editor and analysis program for Windows 95/98/NT. *Nucleic Acids Symposium Series* **41**, 95–98.

Rambaldi D, Ciccarelli FD. 2009. FancyGene: dynamic visualization of gene structures and protein domain architectures on genomic loci. *Bioinformatics* **25**, 2281–2282.

Tuominen LK, Johnson VE, Tsai CJ. 2011. Differential phylogenetic expansions in BAHD acyltransferases across five angiosperm taxa and evidence of divergent expression among *Populus* paralogues. *BMC Genomics* **12**, 236.

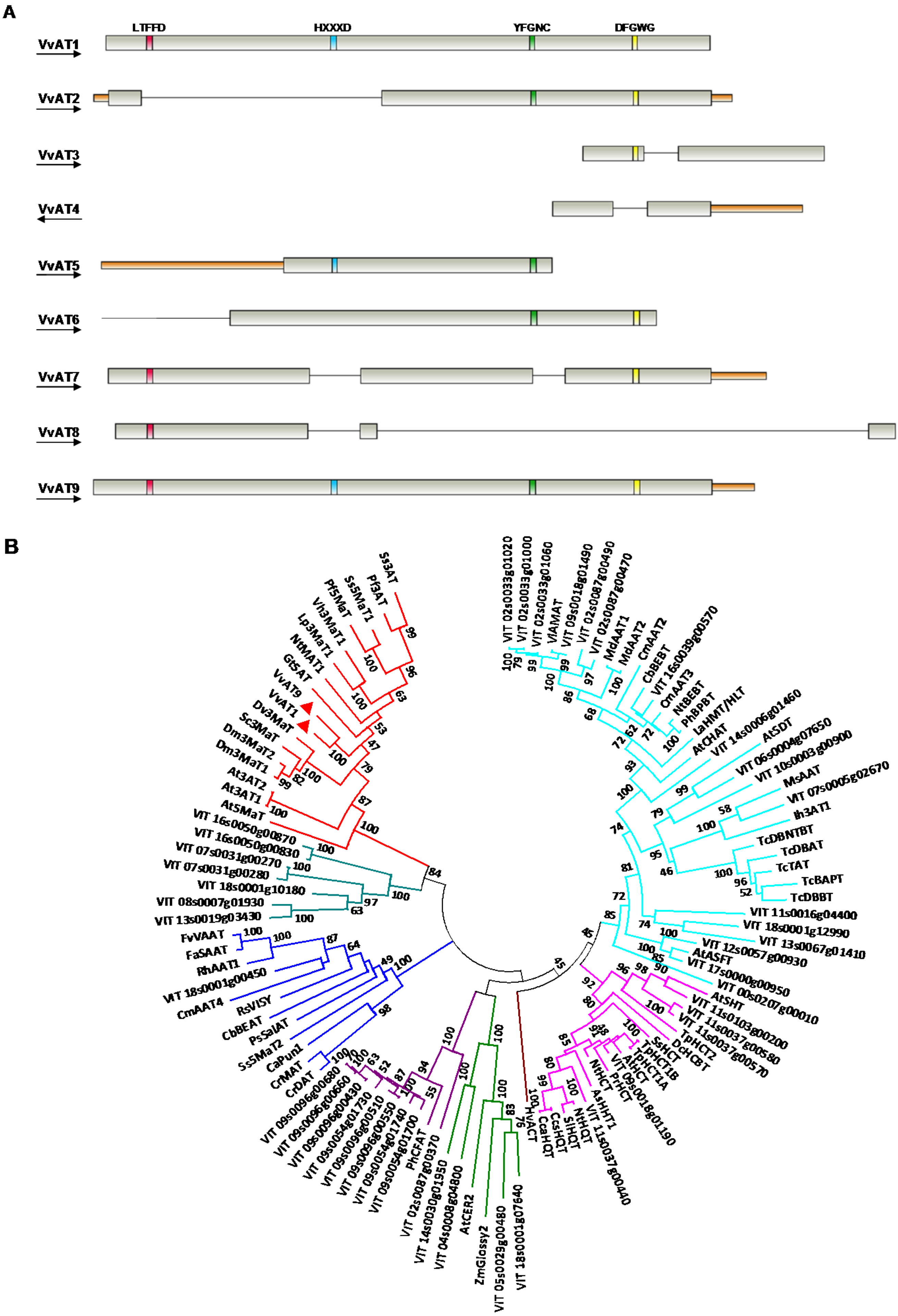
A
LTFFD
HXXXD
YFGNC
DFGWG
VvAT1
VvAT2
VvAT3
VvAT4
VvAT5
VvAT6
VvAT7
VvAT8
VvAT9
B
Ss3AT
Pf3AT
Ss5MaT1
Pf5MaT
Vh3MaT1
Lp3MaT1
NtMAT1
Gt5AT
VvAT9
VvAT1
Dv3MaT
Sc3MaT
Dm3MaT2
Dm3MaT1
At3AT2
At3AT1
At5MaT
VIT 16s0050g00870
VIT 16s0050g00830
VIT 07s0031g00270
VIT 07s0031g00280
VIT 18s0001g10180
VIT 08s0007g01930
VIT 13s0019g03430
FvVAAT
FaSAAT
RhAAT1
VIT 18s0001g00450
CmAAT4
RsVISY
CbBEAT
PsSalAT
Ss5MaT2
CaPun1
CrMAT
CrDAT
VIT 09s0096g00680
VIT 09s0096g00660
VIT 09s0096g00430
VIT 09s0054g01730
VIT 09s0096g00510
VIT 09s0096g00550
VIT 09s0054g01740
VIT 09s0054g01700
PhCFAT
VIT 02s0087g00370
VIT 14s0030g01950
VIT 04s0008g04800
AtCER2
ZmGlossy2
VIT 05s0029g00480
VIT 18s0001g07640
HvACT
CcaHQT
CcsHQT
SlHQT
NtHQT
AsHHT1
VIT 11s0037g00440
NtHCT
PtHCT
AtHCT
VIT 09s0018g01190
TpHCT1A
TpHCT1B
SsHCT
DcHCBT
TpHCT2
VIT 11s0037g00570
VIT 11s0037g00580
VIT 11s0103g00200
AtSHT
VIT 00s0207g00010
VIT 17s0000g00950
AtASFT
VIT 12s0057g00930
VIT 13s0067g01410
VIT 18s0001g12990
VIT 11s0016g04400
TcDBBT
TcBAPT
TcTAT
TcDBAT
TcDBNTBT
Ih3AT1
VIT 07s0005g02670
MsAAT
VIT 10s0003g00900
VIT 06s0004g07650
AtSDT
VIT 14s0006g01460
AtCHAT
LaHMT/HLT
PhBPBT
NtBEBT
CmAAT3
VIT 16s0039g00570
CbBEBT
CmAAT2
MdAAT2
MdAAT1
VIT 02s0087g00470
VIT 02s0087g00490
VIT 09s0018g01490
VlAMAT
VIT 02s0033g01060
VIT 02s0033g01000
VIT 02s0033g01020
